# Patterns of Respiratory Symptoms and Asthma Diagnosis in School‐Age Children: Three Birth Cohorts

**DOI:** 10.1111/all.16617

**Published:** 2025-06-12

**Authors:** Alex Cucco, Angela Simpson, Sadia Haider, Clare Murray, Stephen Turner, Paul Cullinan, Sarah Filippi, Sara Fontanella, Adnan Custovic, STELAR/UNICORN Investigators, Raquel Granell, Graham Roberts, John W. Holloway, Syed Hasan Arshad, Ashley Woodcock, Graham Devereux

**Affiliations:** ^1^ National Heart and Lung Institute Imperial College London London UK; ^2^ Department of Socio‐Economic, Managerial, and Statistical Studies G. d’Annunzio University Chieti‐Pescara Chieti Italy; ^3^ Division of Infection, Immunity and Respiratory Medicine, School of Biological Sciences, Faculty of Biology, Medicine and Health University of Manchester, Manchester Academic Health Science Centre Manchester UK; ^4^ Royal Aberdeen Children's Hospital NHS Grampian Aberdeen UK; ^5^ Department of Mathematics Imperial College London London UK

**Keywords:** asthma, birth cohorts, childhood, clustering, RSV bronchiolitis

## Abstract

**Background:**

Many studies used information on wheeze presence/absence to determine asthma‐related phenotypes. We investigated whether clinically intuitive asthma subtypes can be identified by applying data‐driven semi‐supervised techniques to information on frequency and triggers of different respiratory symptoms.

**Methods:**

Partitioning Around Medoids clustering was applied to data on multiple symptoms and their triggers in school‐age children from three birth cohorts: MAAS (*n* = 947, age 8 years), SEATON (*n* = 763, age 10) and ASHFORD (*n* = 584, age 8). ‘Guided’ clustering, incorporating asthma diagnosis, was used to select the optimal number of clusters.

**Results:**

Five‐cluster solution was optimal. Based on their clinical characteristics, including frequency of asthma diagnosis, we interpreted one cluster as ‘Healthy’. Two clusters were characterised by high asthma prevalence (95.89% and 78.13%). We assigned children with asthma in these two clusters as ‘persistent, multiple‐trigger, more severe’ (PMTS) and ‘persistent, triggered by infection, milder’ (PIM). Children with asthma in the remaining two clusters were assigned as ‘mild‐remitting wheeze’ (MRW) and ‘post‐bronchiolitis resolving asthma’ (PBRA). PBRA was associated with RSV bronchiolitis in infancy. In most children with asthma in this cluster wheezing resolved by age 5–6, and predominant symptoms were shortness of breath and chest tightness. Children in PBRA had the highest hospitalisation rates and wheeze exacerbations in infancy. From age 8 years (cluster derivation) to early adulthood (18–20 years), lung function was significantly lower, and FeNO and airway hyperreactivity significantly higher in PMTS compared to all other clusters.

**Conclusions:**

Patterns of coexisting symptoms identified by semi‐supervised data‐driven methods may reflect pathophysiological mechanisms of distinct subtypes of childhood wheezing disorders.

## Graphical abstract

We performed guided multi‐domain clustering of symptoms and triggers in school‐age children across three birth cohorts. Using 15 variables, five clusters were identified. Two clusters showed a high prevalence of asthma with persistent symptoms. Questions on shortness of breath and chest tightness upon awakening were key to identifying ‘post‐bronchiolitis resolving asthma’. ASHFORD, birth cohort study established in 1991 in Ashford, UK; LRTIs, lower respiratory tract infections; MAAS, Manchester Asthma and Allergy Study; SEATON, The Study of Eczema and Asthma to Observe the influence of Nutrition.

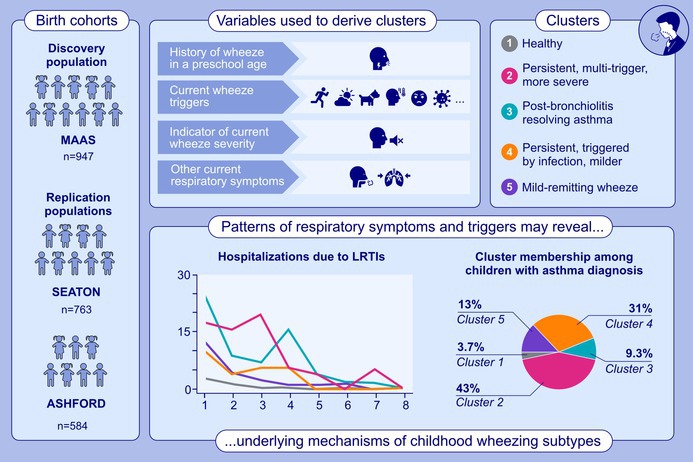

## 1 Introduction

Better understanding of asthma heterogeneity and accurate identification of subgroups of patients in whom symptoms are caused by different mechanisms is important for moving towards personalised treatment [1, 2, 3, 4]. The process of asthma disaggregation can be facilitated using hypothesis‐generating unbiased approaches [5, 6, 7, 8, 9, 10, 11, 12, 13]. Different patterns of symptoms may indicate distinct causes and biological mechanisms [14, 15], and over the past decade, substantial efforts have been devoted to understanding the heterogeneity of childhood asthma by applying data‐driven methodologies [16, 17]. Most studies applied the latent class analysis (LCA) to longitudinal information on wheeze to uncover temporal patterns over a specified time interval [18].

Wheeze phenotypes in different studies are usually designated with the similar/same name, but they often differ in symptom trajectories, prevalence and risk factors [18, 19]. This within‐phenotype heterogeneity and imprecise individual allocation [7, 20, 21] may be partly responsible for a lack of consistent associations of derived phenotypes with risk factors [8]. Other methods which derive more internally homogeneous and robust wheeze phenotypes have recently been reported, but these studies also found that > 5% of adolescents with doctor‐diagnosed asthma never reported wheezing, and a similar proportion had transient wheeze [22]. This highlights the heterogeneity of doctor‐diagnosed asthma and may reflect the fact that children with other respiratory symptoms (even in the absence of wheezing) are diagnosed with asthma [23].

The burden of symptoms reported by patients and/or their carers may capture the subjective elements of disease and is used as an endpoint in clinical trials [24], but is less often used to understand the heterogeneity of asthma. We hypothesise that clinically intuitive subgroups of asthma‐related respiratory conditions which may better reflect underlying mechanisms can be identified by applying data‐driven techniques to detailed information on frequency, severity and triggers of different respiratory symptoms, rather than focusing on wheeze. Despite its potential, this approach has been explored in only a limited number of studies. For example, Spycher et al. [25, 26] used LCA in a population‐based cohort to identify childhood wheeze and chronic cough phenotypes based on multiple disease dimensions, whereas Garden et al. [27] applied latent transition analysis to track asthma phenotype changes over time.

In this work, we also propose that the information on whether individuals have an asthma diagnosis may be of value to the data‐driven discovery of asthma subtypes, since physicians looking after these participants may observe patterns of symptoms that are not captured in the structured questionnaire‐based research follow‐ups. Unlike previous fully unsupervised studies, we used a semi‐supervised approach where asthma diagnosis helps guide clustering. This method retains the data‐driven nature of the analysis while incorporating clinical insight, allowing for a more precise identification of meaningful subgroups and improving their interpretability and relevance [28].

To address our hypotheses, we performed a multi‐domain clustering of data collected at school age in three population‐based birth cohorts. Our study combined information on multiple symptoms (wheeze, shortness of breath and chest tightness), as well as frequency, severity and triggers of symptoms to yield refined asthma phenotypes, potentially reflecting distinct underlying mechanisms. Additionally, we applied ‘guided’ clustering by introducing information on asthma to select the optimal number of clusters. Finally, we investigated early‐life and genetic associates and later‐life objective outcomes (including lung function, allergic sensitisation and airway hyperreactivity‐AHR) of the derived clusters.

## 2 Methods

Detailed methods and definition of all variables are provided in Appendix S1.

### 2.1 Study Design, Setting and Participants

We used data from three UK population‐based birth cohorts. Discovery analyses were carried out in the Manchester Asthma and Allergy Study (MAAS) [29], and replication in Ashford [30] and Aberdeen (SEATON) cohorts [31]. All studies recruited pregnant women who gave birth to 1184, 642 and 1924 children respectively, and were approved by research ethics committees. Informed consent was obtained from parents. Data were integrated to facilitate joint analyses [32].

### 2.2 Data Sources

For cluster derivation we used data collected at age 8 years in MAAS and ASHFORD, and at age 10 in SEATON. Validated questionnaires [33] were administered to collect information on parentally reported symptoms, physician‐diagnosed illnesses and medication usage.

We used objective outcomes for cluster validation. Allergic sensitisation was assessed through skin prick tests. Lung function was measured using spirometry, and AHR using methacholine challenge (MAAS only) [34]. Fractional exhaled Nitric Oxide (FeNO) was measured as a marker of airway inflammation [35].

### 2.3 Variables Used to Derive Clusters

We used 15 variables to derive clusters:

- *History of wheeze in pre‐school age:* (1) wheeze ever to age 5 years.
- *Current wheeze triggers:* (2) with exercise; (3) without exercise; (4) with colds; (5) apart from colds; (6) weather; (7) pollen; (8) flu; (9) dust; (10) pets; (11) fumes; (12) emotions.
- *Indicator of current wheeze severity:* (13) wheeze limiting speech.
- *Other current respiratory symptoms:* (14) shortness of breath; (15) chest tightness.

The cohort‐specific questions are shown in Table S1.

*Asthma:* Defined as a positive answer to at least two of: ‘Has the doctor ever told you that your child had asthma?’, ‘Has your child had wheezing or whistling in the chest in the last 12 months?’ and ‘Has your child had asthma treatment in the last 12 months?’ [36].

### 2.4 Definition of Objective Outcomes Used for Cluster Validation

*Allergic sensitisation:* Wheal ≥ 3 mm than the negative control to at least one allergen.

*Lung function:* FEV_1_/FVC ratio.

*Airway inflammation:* FeNO (ppb).

*AHR:* ≥ 20% decrease in FEV_1_ by the last dose of the methacholine challenge (16 mg/mL) [11].

### 2.5 Early‐Life Risk Factors, Bronchiolitis, Severe Wheeze Exacerbations and Hospital Admissions

*Early‐life risk factors:* Sex, maternal and paternal smoking, parental history of asthma, hay fever, eczema, sibling with asthma, eczema or hay fever, pet ownership before age 5 years.

In MAAS, we extracted data from primary care medical records, including information on bronchiolitis (Respiratory Syncytial Virus‐RSV confirmed or not), severe wheeze exacerbations [37, 38] and lower respiratory tract infections (LRTI) hospital admissions. This information was available for the first 8 years (before the time of cluster derivation) [39].

### 2.6 Long‐Term Outcomes

To ascertain long‐term outcomes in different clusters in the discovery population, we derived trajectories of lung function, AHR, airway inflammation (FeNO) and sensitisation after cluster derivation (age 8) to early adulthood (ages 11, 16 and 18–20 years).

### 2.7 Statistical Analysis

To identify clusters, we adopted Partition Around Medoids algorithm [40], which is particularly suitable for categorical data due to its medoid‐based approach. We employed a ‘guided’ clustering method, incorporating child's asthma diagnosis to inform the optimal number of clusters. This approach acknowledges that certain symptom patterns observed by physicians might extend beyond the scope of structured questionnaire data, thereby enhancing the clinical relevance and statistical robustness of the identified clusters. Along with classic internal validity measures (the silhouette and *C*‐index), we also adopted the homogeneity measure [41]. The homogeneity measure was applied to evaluate the alignment of clusters with clinically relevant outcomes, specifically doctor‐diagnosed asthma. This ensured that the identified clusters were not only statistically valid but also reflected meaningful phenotypic distinctions related to asthma. While multiple validation metrics were considered, the homogeneity index was particularly relevant for maintaining cluster coherence and helped guide the final choice when discrepancies in the optimal cluster number arose. To reduce the influence of the random initialisation, we repeated the analysis using three different starting points and selected the solution also considering the variability of the performance measures in different repetitions. Further methodological details on the clustering procedures and validation measures are provided in Supporting Information.

#### 2.7.1 Association of Clusters With Early‐Life Risk Factors and Objective Outcomes

Kruskal–Wallis or Wilcoxon test for continuous variables, and Fisher exact or Chi‐squared test for categorical variables.

#### 2.7.2 Genetic Associates of the Derived Clusters

In a candidate gene analysis, we investigated the association of the derived clusters with genetic variants which were previously associated with persistent wheezing and childhood‐onset asthma. We selected three single nucleotide polymorphisms (SNPs) in 17q12‐q21 (rs7216389, rs4795408 and rs3894194) [42, 43, 44, 45], one in *CDHR3* (rs6967330) [46] and two in *Annexin 1* (rs116849664 and rs75260654) [47]. We evaluated the association between SNPs and the clustering solution using Fisher exact test.

## 3 Results

### 3.1 Discovery Population: MAAS

The analysis was conducted in 947/1030 (91.94%) children who attended the follow‐up at age 8 years, for whom complete information about the variables used for clustering was available. Of those, 162 (17.11%) had asthma. Demographic characteristics are shown in Table S2, and the frequency distribution of the 15 variables used for cluster derivation is shown in Table S3.

#### 3.1.1 Derived Clusters

**FIGURE 1:**
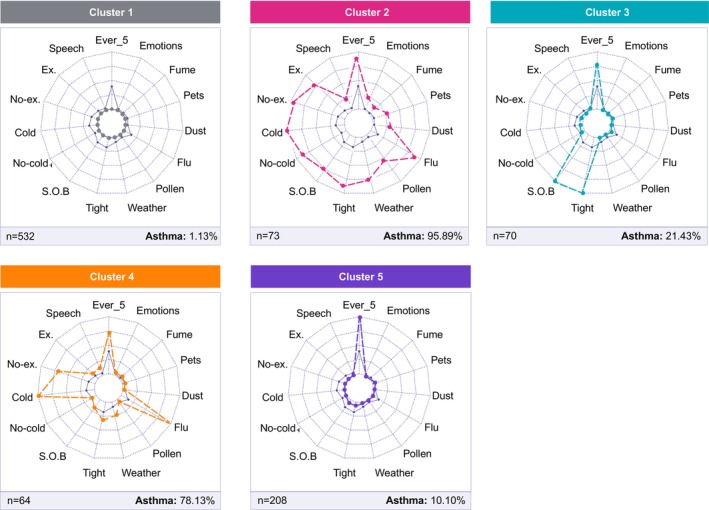
Distribution of the 15 variables across the five clusters retrieved in the MAAS cohort. The position of each dot represents the percentage of children with a positive answer to the associated question for a specific cluster. Each dot is positioned on an axis with range limits 0% and 100%. The distribution of variables in the full dataset is depicted in blue. Ever5: history of wheeze by age 5 years; Ex.: wheeze with exercise; No‐Ex.: wheeze without exercise; Cold: wheeze with colds; No‐Cold: wheeze apart from colds; Wheeze triggers (weather, pollen, flu, dust, pets, fumes and emotions); Speech: wheeze limiting speech; S.O.B: shortness of breath; Tight: tightness in the chest.

A five‐cluster solution was selected as optimal and was stable in relation to the algorithm initialisation (Figure S1). To investigate the characteristics of the clusters, we inspected the distributions of variables included in the clustering procedure, and the distribution of asthma (Figure 1):

*Cluster 1* (C1; 532/947, 56.18%) was characterised by the lowest percentage of children with asthma (*n* = 6, 1.13%), and for most children in this cluster, negative answers were recorded to all 15 questions.

*Cluster 2* (C2; 73/947, 7.71%) had the highest prevalence of asthma (95.89%, 70/73). This cluster was characterised by a high frequency of subjects reporting current wheeze with and apart from colds and other symptoms. Wheeze was caused by a variety of triggers (exercise, flu, emotions, exposure to pollen, dust, pets and fumes; Figure 1). This cluster had the highest proportion of children reporting wheeze limiting speech (15/73, 20.55%). This severity marker was reported by a total of 27/947 (2.85%) of participants, 15 (55.6%) of whom were assigned to C2.

*Cluster 4* (C4; 64/947, 6.76%) also had a high proportion of children with asthma (78.13%; 50/64); in contrast to C2, a high proportion of children in C4 reported wheezing related to cold and flu, but not other triggers or other symptoms (Figure 1).

*Cluster 3* (C3; 70/947, 7.39%) and *Cluster 5* (C5; 208/947, 21.96%) had a lower proportion of children with asthma (21.43% [15/70] and 10.1% [21/208]). The pattern of respiratory symptoms among children in C3 differed from other clusters and was uniquely characterised by shortness of breath and tightness in the chest upon waking.

#### 3.1.2 Characteristics of Clusters

Comparison of children across the clusters is shown in Table 1, Table S4 and Figure S2. Among the two clusters with the highest proportion of children with asthma, those in C2 had significantly higher FeNO at age 8 years compared to C4 (Figure S2a, *p* = 0.03). This cluster was also characterised by significantly lower lung function (Figure S2b) and higher frequency of allergic sensitisation (Table 1). C2 also had the highest proportion of positive methacholine challenges (Table 1).

**TABLE 1:** Characteristics of children in five clusters.

		Cluster 1, 532/947 (56.18%)	Cluster 2, 73/947 (7.71%)	Cluster 3, 70/947 (7.39%)	Cluster 4, 64/947 (6.76%)	Cluster 5, 208/947 (21.96%)	Effect size, Cramer's *V* ^a^	*p*, Fisher test
Asthma diagnosed (yes)		1.13% 6/532	95.89% 70/73	21.43% 15/70	78.13% 50/64	10.10% 21/208	0.790	< 0.0001
Current wheeze age 7–8		1.32% 7/532	100.00% 73/73	10.00% 7/70	100.00% 64/64	4.33% 9/208	0.914	< 0.0001
Wheeze ever to age 5 years		0.00% 0/532	89.04% 65/73	78.57% 55/70	70.31% 45/64	100.00% 208/208	0.926	< 0.0001
**Wheeze triggers**								
Wheeze with exercise		0.75% 4/532	73.97% 54/73	0.00% 0/70	12.50% 8/64	1.44% 3/208	0.750	< 0.0001
Wheeze without exercise		0.19% 1/532	90.41% 66/73	4.29% 3/70	67.19% 43/64	1.44% 3/208	0.856	< 0.0001
Wheeze with colds		0.38% 2/532	97.26% 71/73	4.29% 3/70	98.44% 63/64	0.96% 2/208	0.958	< 0.0001
Wheeze apart from colds		1.13% 6/532	83.56% 61/73	5.71% 4/70	7.81% 5/64	1.92% 4/208	0.784	< 0.0001
Wheeze with changes in weather		0.00% 0/532	76.71% 56/73	0.00% 0/70	25.00% 16/64	1.44% 3/208	0.771	< 0.0001
Wheeze with exposure to pollen		0.19% 1/532	56.16% 41/73	0.00% 0/70	4.69% 3/64	0.48% 1/208	0.692	< 0.0001
Wheeze with flu		0.00% 0/532	93.15% 68/73	4.29% 3/70	96.88% 62/64	0.96% 2/208	0.949	< 0.0001
Wheeze with exposure to dust		0.00% 0/532	34.25% 25/73	0.00% 0/70	3.13% 2/64	0.48% 1/208	0.536	< 0.0001
Wheeze with exposure to pets		0.19% 1/532	31.51% 23/73	4.29% 3/70	4.69% 3/64	1.92% 4/208	0.441	< 0.0001
Wheeze with exposure to fumes		0.00% 0/532	16.44% 12/73	0.00% 0/70	4.69% 3/64	0.00% 0/208	0.356	< 0.0001
Wheeze triggered by emotions		0.00% 0/532	24.66% 18/73	0.00% 0/70	4.69% 3/64	0.48% 1/208	0.435	< 0.0001
**Wheeze severity (age 8)**								
Wheeze limiting speech		0.19% 1/532	20.55% 15/73	2.86% 2/70	14.06% 9/64	0.00% 0/208	0.372	< 0.0001
**Other symptoms (age 8 years)**								
Shortness of breath		1.50% 8/532	72.60% 53/73	100.00% 70/70	18.75% 12/64	5.77% 12/208	0.815	< 0.0001
Tightness of the chest		1.13% 6/532	87.67% 64/73	100.00% 70/70	31.25% 20/64	5.77% 12/208	0.851	< 0.0001
**Demographic/early life characteristics**								
Sex (female)		52.63% 280/532	34.25% 25/73	32.86% 23/70	37.50% 24/64	40.38% 84/208	0.155	0.0001
Maternal asthma ever (yes)		15.60% 83/532	23.29% 17/73	24.29% 17/70	21.88% 14/64	27.40% 57/208	0.126	0.004
Maternal current asthma (yes)		10.34% 55/532	16.90% 12/71	17.14% 12/70	20.31% 13/64	21.26% 44/207	0.135	0.001
**LRTI hospitalisations in the first 8 years of life**		5.16% 24/465	45.61% 26/57	44.83% 26/58	21.43% 12/56	20.99% 38/181	0.337	< 0.0001
**Bronchiolitis**								
Confirmed bronchiolitis		3.01% 14/465	10.53% 6/57	15.52% 9/58	5.36% 3/56	7.73% 14/181	0.159	0.0004
Confirmed respiratory syncytial virus (RSV)‐positive bronchiolitis		0.86% 4/465	5.26% 3/57	10.34% 6/58	3.56% 2/56	4.42% 8/181	0.163	0.0003
**Wheeze severity**								
Non‐wheezers		98.68% 525/532	0 0/73	90% 63/70	0% 0/64	95.67% 199/208	0.914	< 0.0001
Mild wheezers		0.75% 4/532	31.51% 23/73	5.71% 4/70	73.44% 47/64	3.37% 7/208	0.669	
Moderate/severe wheezers		0.56% 3/532	68.49% 50/73	4.29% 3/70	26.56% 17/64	0.96% 2/208	0.691	
**Polymorphisms in 17q21 and CDHR3**		*N* = 489	*N* = 69	*N* = 59	*N* = 56	*N* = 188		
rs7216389	CC	28.43%	14.49%	35.59%	23.21%	29.79%	0.086	0.09
	CT	48.67%	56.52%	37.29%	42.86%	44.15%		
	TT	22.90%	28.99%	27.12%	33.93%	26.06%		
rs4795408	GG	34.36%	17.39%	37.29%	33.93%	36.70%	0.093	0.04
	GA	46.01%	59.42%	47.46%	39.29%	38.83%		
	AA	19.63%	23.19%	15.25%	26.79%	24.47%		
rs3894194	GG	34.36%	17.39%	35.59%	32.14%	37.23%	0.090	0.07
	GA	46.01%	62.32%	47.46%	41.07%	40.43%		
	AA	19.63%	20.29%	16.95%	26.79%	22.34%		
rs6967330	GG	71.17%	55.07%	69.49%	58.93%	69.15%	0.112	0.01
	GA	26.99%	37.68%	30.51%	35.71%	30.32%		
	AA	1.84%	7.25%	0.00%	5.36%	0.53%		
**Polymorphisms in ANAXA1**		*N* = 460	*N* = 67	*N* = 57	*N* = 54	*N* = 185		
rs116849664_T	CC	98.04%	91.04%	96.49%	98.15%	96.22%	0.103	0.04
	TC	1.96%	7.46%	3.51%	1.85%	3.78%		
	TT	0.00%	1.49%	0.00%	0.00%	0.00%		
rs75260654_T	CC	96.96%	89.55%	96.49%	96.30%	97.30%	0.102	0.08
	TC	3.04%	8.96%	3.51%	3.70%	2.70%		
	TT	0.00%	1.49%	0.00%	0.00%	0.00%		

^a^ Reference values for Cramer's *V* association are: *V* ≤ 0.10 = weak association (for df = 1; lower thresholds if df > 1); 0.10 < *V* ≤ 0.30 = moderate association; *V* > 0.30 = strong association.

We categorised children as non‐wheezers, mild wheezers and moderate/severe wheezers (definitions in Supporting Information). Among clusters with a high proportion of children with asthma, the proportion of those with moderate/severe wheeze was much higher in C2 (68.49%) than in C4 (26.56%) (Table 1, *p* < 0.0001).

There were significant differences between clusters in *GSDMA* SNP rs4795408 (*p* = 0.04), *CDHR3* SNP rs6967330 (*p* = 0.01) and *ANAXA1* SNP rs116849664 (*p* = 0.04) (Table 1), with asthma risk alleles being overrepresented in C2 and C4.

#### 3.1.3 Cluster Membership Among Children With Asthma Diagnosis

**FIGURE 2:**
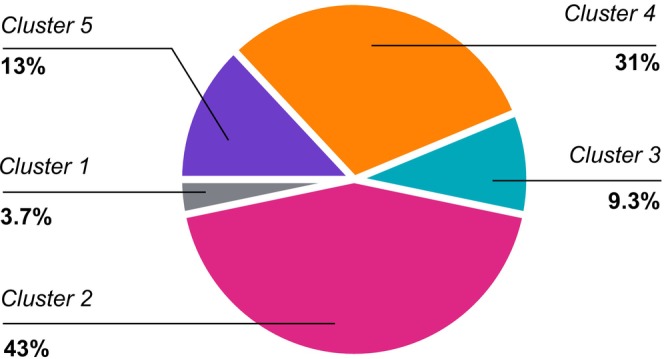
Children with asthma diagnosis (*n* = 162) in the discovery population (Manchester Asthma and Allergy Study): Absolute frequency across the five clusters. C1: ‘Healthy’ (*n* = 6); C2: ‘Persistent, multiple‐trigger, more severe asthma’ (*n* = 70); C3: ‘Post‐bronchiolitis resolving asthma’ (*n* = 15); C4: ‘Persistent, triggered by infection, milder asthma’ (*n* = 50); C5: ‘Mild‐remitting wheeze’ (*n* = 21).

Among 162 children with asthma, 70 (43%) were in C2, 50 (31%) in C4, 21 (13%) in C5, 15 (9.3%) in C3 and 6 (3.7%) in C1 (Figure 2). All children with asthma in C2 and C4 reported current wheezing at age 8 years, and severity patterns were similar to those described above. In contrast, most children with asthma in C3 (10/15, 66.7%) and C5 (13/21, 61.9%) did not report current wheeze, but only wheezing in pre‐school (Table 2).

**TABLE 2:** Current wheeze at age 8 years, severe wheeze exacerbation with oral corticosteroids receipt in the first 8 years of life and in the eighth year among children with asthma diagnosis across the five clusters.

	Cluster 1: Healthy, 6/532	Cluster 2: PMTS, 70/73	Cluster 3: PBRA, 15/70	Cluster 4: PIM, 50/64	Cluster 5: MRW, 21/208	Effect size, Cramer's *V* ^a^	*p*, Fisher test
Current wheeze (8 years)	66.67% 4/6	100% 70/70	33.33% 5/15	100% 50/50	38.10% 8/21	0.803	< 0.0001
Severe wheeze exacerbation with oral corticosteroids receipt from birth to age 8	0% 0/5	53.7% 29/54	75% 9/12	38.1% 16/42	31.58% 6/19	0.305	0.01
Severe asthma exacerbation (8th year of life)	0.00% 0/5	12.96% 7/54	0.00% 0/12	7.14% 3/42	5.26% 1/19	0.160	0.72

*Note:* The head of each column displays the number of children with asthma within the total cluster size; the percentage reported in the table is obtained by computing the proportion of reported symptoms among children with asthma in each group for whom the associated information was available. C1: ‘Healthy’; C2: ‘Persistent, multiple‐trigger, more severe asthma’ (PMTS); C3: ‘Post‐bronchiolitis resolving asthma’ (PBRA); C4: ‘Persistent, triggered by infection, milder asthma’ (PIM); and C5: ‘Mild‐remitting wheeze’ (MRW).

^a^ Reference values for Cramer's *V* association are: *V* ≤ 0.10 = weak association (for df = 1; lower thresholds if df > 1); 0.10 < *V* ≤ 0.30 = moderate association; *V* > 0.30 = strong association.

#### 3.1.4 Bronchiolitis

The proportion of children with confirmed bronchiolitis and RSV‐positive bronchiolitis differed significantly between the clusters and was highest in C3 (Table 1).

#### 3.1.5 Severe Wheeze Exacerbations and LRTIs Hospitalisations in the First 8 Years

**FIGURE 3:**
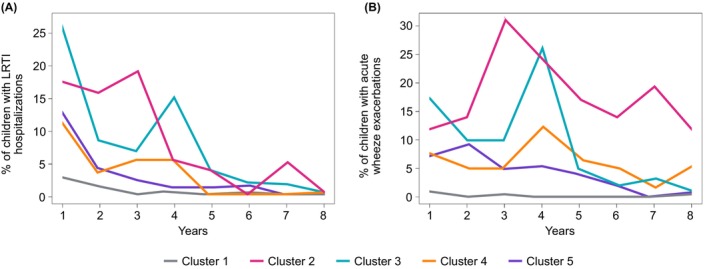
Hospital admissions due to the lower respiratory tract infections (LRTIs) and acute severe wheeze exacerbations in the first 8 years of life (prior to cluster derivation); information ascertained from the primary care health records. (A) Percentages of children with LRTI hospitalisation. (B) Percentages of children with acute severe wheeze exacerbation.

Patterns of wheeze attacks and LRTIs hospitalisations in the first 8 years (prior to cluster derivation) are shown in Figure 3. Children in C3 had the highest rate of LRTI hospitalisations in infancy, and C2 and C3 in pre‐school age (Figure 3A), with no differences between the clusters in school age. Severe exacerbations were most common in C2 from infancy to age 8 years (Figure 3B). Among children with asthma, severe exacerbations with oral corticosteroid receipt were most common in C3 (9/12; 75%). However, most of these occurred in early life, and none of these children had exacerbations in the 8th year (Table 2).

#### 3.1.6 Long‐Term Outcomes: Lung Function, Sensitisation and AHR From Age 8 to Age 18–20 Years

Figures S3–S5 show the trajectories of lung function, FeNO, sensitisation and AHR across the clusters from age 8 years (cluster derivation) to early adulthood (age 18–20 years). Lung function was significantly lower, FeNO higher and the proportion of sensitised individuals and those with AHR significantly higher in C2 compared to all others.

#### 3.1.7 Cluster Interpretation

Based on cluster characteristics presented above, we interpreted C1 as ‘Healthy’. We assigned children with asthma in other four clusters as: C2—‘Persistent, multiple‐trigger, more severe asthma’; C4—‘Persistent, triggered by infection, milder asthma’; C3—‘Post‐bronchiolitis resolving asthma’; and C5—‘Mild‐remitting wheeze’.

### 3.2 External Validation

#### 3.2.1 SEATON

We analysed data from 763/1924 participants, of whom 81 (10.6%) had asthma at age 10. We used questions representing six different domains: wheeze ever, wheeze with cold, wheeze without cold, speech limited by wheeze, ever woken with shortness of breath and/or with tightness in the chest. Although the information was less granular compared to MAAS, a similar five‐cluster solution was optimal (Figure S6). Figure S7 shows the distribution of variables we used to derive clusters. Comparisons of children across the five clusters are shown in Table S5. Within‐cluster patterns (Figure S7) and their associates (Table S5), including FeNO and sensitisation (Figure S8) were consistent with those in MAAS.

Analyses among 744 children with complete data (Figures S9 and S10) and after cross‐checking of questions to limit inconsistencies (Figures S11 and S12) provided remarkably consistent results.

#### 3.2.2 Ashford

We analysed data from 584/642 children, of whom 70 (12%) had asthma at age 8. Unlike MAAS and SEATON, in ASHFORD the information related to shortness of breath and chest tightness was not collected, but wheeze triggers were available (Table S1). The distance measure was computed along nine dimensions: wheeze ever, wheeze with flu, wheeze without flu, speech limited by wheeze, wheeze triggered by dust, pets, weather, emotions and exercise.

A four‐clusters solution was optimal (Figure S13). The characterisation of each cluster highlights the similarities and differences with the solutions in MAAS and SEATON (Figure S14). C1 mainly contained children who never wheezed. Similar to the other two cohorts, we identified two clusters with a high proportion of children with asthma (C2 and C4). As in other cohorts, C2 was characterised by a high frequency of subjects reporting wheeze with a variety of triggers. This cluster was also characterised by the highest proportion of children reporting severe wheeze. In C4, a high proportion of children reported wheezing related to colds, but not other triggers. C3 comprised mainly children who wheezed before age 5 but had no current wheeze at age 8 years (Table S6).

### 3.3 Impact of Different Questions on Optimal Solution

**FIGURE 4:**
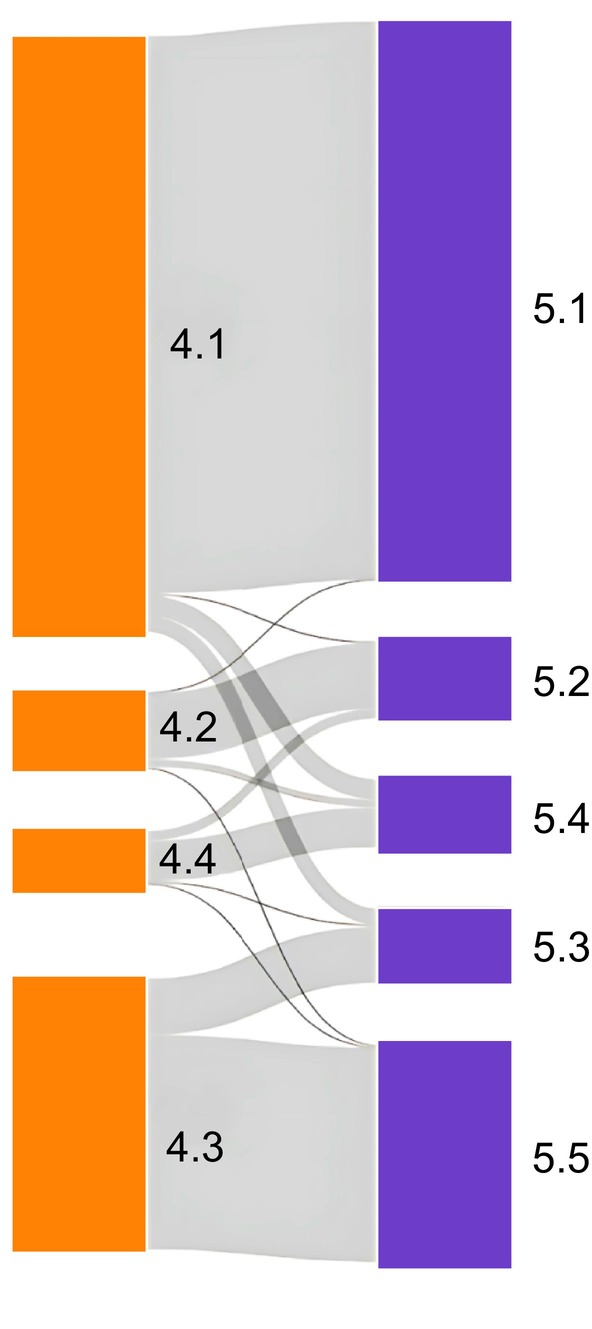
Comparison between the partition obtained using the restricted set of variables and all variables in the MAAS cohort. On the left‐hand side, the four‐cluster solution retrieved using the restricted set of variables also available in Ashford is displayed (clusters 4.1, 4.2, 4.3 and 4.4). The five‐cluster solution retrieved using the complete set of variables is displayed on the right (5.1, 5.2, 5.3, 5.4 and 5.5). The key difference to the results using the complete dataset was that C3 and C5 merged into a single cluster.

To understand the impact of different questions on the optimal solution, we repeated the analysis in MAAS using only the information available in Ashford. Using this subset of data, a four‐cluster solution was optimal (Figure S15), with highly consistent clusters compared to Ashford (Figure S16). The key difference to the results using the complete dataset was that C3 and C5 merged into a single cluster (Figure 4).

### 3.4 Feature Importance in Deriving Clusters

We ascertained the importance of individual variables in the identification of individual clusters (Supporting Information, Table S7). The history of pre‐school wheezing, the cold/flu trigger, the shortness of breath and chest tightness represent the most important symptoms to derive the described clusters.

## 4 Discussion

### 4.1 Key Findings

We performed a multi‐domain clustering of several participant‐reported symptoms, their triggers and indicators of severity collected in three birth cohorts. We utilised innovative methodology of guided clustering which introduced the information on asthma diagnosis to inform the number of clusters. In the discovery population, we identified five clusters by using 15 variables. The percentages of children with asthma differed substantially between the clusters, ranging from ~1% to 96%. Two clusters were characterised by a very high proportion of children with asthma. Although these clusters shared some similar clinical features such as allergic sensitisation, the pattern of symptoms differed: one was characterised only by wheezing related to infection (C4), and the other by the presence of other symptoms and multiple triggers of wheezing (C2). The latter group contained children with more frequent and severe symptoms. This was also reflected in a significantly higher FeNO, lower lung function and higher proportion of children with AHR in C2.

Participants who reported pre‐school wheeze and shortness of breath and chest tightness in the school age clustered in a distinct subgroup (C3), which was characterised by the highest hospitalisation and exacerbation rates in the first year of life and the highest percentage of children who had RSV‐confirmed bronchiolitis.

We estimated the relative contribution of different clusters to the overall asthma diagnosis. Among children with asthma in mid‐school age, ~43% have persistent, multiple‐trigger, more symptomatic asthma; ~31% have persistent milder asthma, triggered mostly by infection; ~9%–10% have post‐bronchiolitis resolving asthma and the remaining 16%–17% have mild transient wheeze.

In Ashford cohort, in which the information about the shortness of breath and chest tightness was missing, a four‐cluster solution was retrieved: ‘Healthy’, ‘Transient wheeze’ and two persistent asthma groups. Further analyses demonstrated that the questions on shortness of breath and chest tightness were essential to identify C3. In the absence of these questions, this cluster merged with C5 (mild‐remitting wheeze) to form a single ‘Transient wheeze’ cluster characterised by the resolution of mostly infection‐induced wheezing by age 5–8 years.

### 4.2 Limitations and Strengths

Our study has strengths and limitations. We acknowledge the complexity of the analyses and the subtle differences in question wording between cohorts. However, results were remarkably consistent across the cohorts.

We could not ascertain the impact of cough, as there was a very high correlation between the response to the questions on cough with those on wheeze. While using cough questions in the clustering procedure might be tempting, the observed correlation between the two symptoms would lead to unstable clusters, and we opted to retain information on wheeze.

The names of the derived clusters and potential underlying mechanisms need to be interpreted with caution. For example, while C3 had the highest percentage of confirmed RSV bronchiolitis, this was nonetheless relatively low. However, other characteristics of this cluster, including the highest hospitalisation and wheeze exacerbation rates in infancy, point towards the importance of early‐life susceptibility to RSV infection. Also of note, it is highly unlikely that the described clusters are underpinned by completely unique mechanisms, and children with RSV bronchiolitis were also present in other clusters. It is therefore possible that RSV has contributed to the development of asthma in other clusters as well, possibly in interaction with other pathways (e.g., T2 mechanisms).

We acknowledge the potential impact of asthma medication on the reported symptoms. We accounted for the possible confounding effects of medication use by integrating this information in our analysis (medications use was utilised to define asthma, which was used in a guided clustering to select the optimal number of clusters).

Our approach is hypothesis generating, and further analyses in cohorts with similar information and at different ages are required to validate the structure. However, our findings suggest that utilising comprehensive information on reported symptoms, which includes life‐course history of wheeze, specific wheeze triggers and other symptoms (specifically shortness of breath and tightness of the chest), can provide clues about specific asthma subtypes. In our analysis, history of pre‐school wheezing, current wheeze with or apart from colds and current shortness of breath and chest tightness were the most important symptoms to differentiate the clusters. While it might be tempting to obtain a predictive algorithm that allocates individuals into specific clusters, we emphasise the explorative nature of an unsupervised analysis. Although it is sensible to investigate the importance of a variable used to derive a partition and speculate about the role of specific features in determining different phenotypic expression, the unsupervised nature of the study should be considered when interpreting the results.

### 4.3 Interpretation

We propose that among children with asthma, there may be four distinct subgroups which can be ascertained through careful clinical history. Doctors should be alerted by the report of multiple triggers of wheezing and other symptoms, as this pattern may be associated with more troublesome disease. Children reporting wheeze mostly with colds, although as frequently sensitised as those with multiple triggers, tend to have milder disease.

We identified a subgroup of children in whom asthma appears to be predominantly associated with early‐life bronchiolitis. This group accounts for ~10% of those with asthma in school age. The longitudinal patterns of exacerbations indicated the validity of this cluster, with those in post‐bronchiolitis asthma having a high burden in early life, upon which there was a substantial reduction in attacks by mid‐school age. In contrast, among those in the asthma cluster characterised by multiple triggers, exacerbation frequency increased through the first 3 years and remained high thereafter. This is important, since many observational studies have reported an association between RSV bronchiolitis and subsequent recurrent wheezing and asthma among school‐age children [48, 49, 50]. There are major advances in RSV prevention, including palivizumab for prophylaxis of severe RSV infections in high‐risk children [51], a long‐acting monoclonal antibody nirsevimab for prevention of RSV in infants during their first RSV season [52] and maternal vaccination for prevention of RSV in infants [53], raising an important question of whether prophylaxis against RSV in early life may reduce the risk of subsequent asthma. Our results should be considered in the design of future studies addressing this important question. We would anticipate that the impact of such interventions would potentially be substantial but limited to a specific subtype of childhood asthma, and potentially much greater in the developing countries [54]. Studies designed to ascertain the impact of such interventions on ‘recurrent wheeze’ or ‘doctor‐diagnosed asthma’ may be underpowered due to the heterogeneity of these outcomes and may underestimate the potentially considerable effect in a subgroup.

In conclusion, patterns of different respiratory symptoms and their triggers identified by semi‐supervised data‐driven methods may reflect pathophysiological mechanisms of distinct subtypes of childhood wheezing disorders.

## Author Contributions

A.Cucco, S.F. and A.Custovic conceived and planned the study, and wrote the manuscript. A.Cucco and S.F. analysed the data. All authors contributed to the interpretation of the results. All authors provided critical feedback and helped shape the research, analysis and manuscript.

## Disclosure

This work forms part of a submitted PhD thesis, which will be publicly available in the Imperial College repository, Spiral, under a CC BY‐NC licence after a minimum 2‐year embargo period (extendable).

## Conflicts of Interest

Dr. Custovic reports personal fees from Sanofi and personal fees from Reacta Healthcare outside the submitted work. Dr. Simpson reports lecture fees from Chiesi. Dr. Murray reports personal fees from Novartis, grants and personal fees from GSK, personal fees from Boerhinger Ingelheim. Other authors have no competing interests to declare.

## Supporting information


Appendix S1

## Data Availability

An anonymised, de‐identified version of the dataset can be made available on request to allow all results to be reproduced. All requests should be directed to the STELAR Principal Investigators.

## Appendix 1 Stelar Investigators

Raquel Granell^1^, Graham Roberts^2,3,4^, John W. Holloway^3,4^, Syed Hasan Arshad^2,3,4^, Ashley Woodcock^5^, Graham Devereux^6^

^1^MRC Integrative Epidemiology Unit, Population Health Sciences, Bristol Medical School, University of Bristol, UK

^2^Human Development and Health and 10Clinical and Experimental Sciences, Faculty of Medicine, University of Southampton, Southampton, UK

^3^National Institute for Health Research Southampton Biomedical Research Centre, University Hospitals Southampton National Health Service Foundation Trust, Southampton, UK

^4^David Hide Asthma and Allergy Research Centre, Isle of Wight, UK

^5^Division of Infection, Immunity and Respiratory Medicine, School of Biological Sciences, Faculty of Biology, Medicine and Health, University of Manchester, Manchester Academic Health Science Centre, Manchester, UK

^6^Clinical Sciences, Liverpool School of Tropical Medicine, Liverpool, UK
