# Supplementary material for: Patterns of Respiratory Symptoms and Asthma Diagnosis in School‐Age Children: Three Birth Cohorts
Source: Allergy. 2025 Jun 12;80(7):1923–34. doi: 10.1111/all.16617 (PMC12261878; doi:10.1111/all.16617)
Supplement: Supplementary file 1 — Appendix S1 [file ALL-80-1923-s001.docx]

**Patterns of respiratory symptoms and asthma diagnosis in school-age children: Three birth cohorts**

Alex Cucco, Angela Simpson, Sadia Haider, Clare Murray, Stephen Turner, Paul Cullinan, Sarah Filippi, Sara Fontanella, Adnan Custovic

on behalf of STELAR/UNICORN investigators

**SUPPLEMENTARY APPENDIX**

**SUPPLEMENTARY METHODS**

**Data sources and definitions of variables: Description of cohorts**

MAAS

MAAS is an unselected birth cohort study established in 1995 in Manchester, UK.^1^ It consists of a mixed urban-rural population within 50 square miles of South Manchester and Cheshire, United Kingdom located within the maternity catchment area of Wythenshawe and Stepping Hill Hospitals. All pregnant women were screened for eligibility at antenatal visits (8-10^th^ week of pregnancy). Of the 1499 couples who met the inclusion criteria (≤10 weeks of pregnancy, maternal age ≥18 years, and questionnaire and skin prick data test available for both parents), 288 declined to take part in the study and 27 were lost to follow-up between recruitment and the birth of a child. A total of 1184 children were born into the study between February 1996 and April 1998. Participants attended follow-up clinic at age 8 years, which included lung function measurements, skin prick testing, biological samples (serum, plasma and urine), and questionnaire data collection. The study was approved by the North West – Greater Manchester East Research Ethics Committee.

*Data from primary care medical records:* We extracted data from electronic and paper-based primary care medical records, including emergency department admissions, and admissions to hospital. Age in days at the time of each event was documented.^2^ This data was available from birth to age 8 years.

### ASHFORD

The Ashford study is an unselected birth cohort study established in 1991 in Ashford, UK.^3^ It included 642 children born between 1992 and 1993. Participants were recruited prenatally and followed prospectively. For the current analysis, we used data collected at age 8 years, when detailed standardised questionnaires were administered to collect information on the natural history of asthma and other allergic diseases. Lung function measurements and SPT was also carried out.

### SEATON

The Study of Eczema and Asthma to Observe the influence of Nutrition (SEATON) is an unselected birth cohort study established in 1997 in Aberdeen, UK, which was designed to explore the relationship between antenatal dietary exposures and asthma outcomes in childhood^4^. 2000 healthy pregnant women attending an antenatal clinic, at median 12 weeks gestation, were recruited. An interviewer administered a questionnaire to the women and atopic status was ascertained by skin prick test (SPT). The cohort included 1924 children born between April 1998 and December 1999. Participants were recruited prenatally and followed up by self−completion questionnaires. Lung function measurements and SPT to common allergens was performed at age 10 years. The study was approved by the North of Scotland Research Ethics Committee.

**Lung function measurement**

MAAS: Spirometry was performed according to American Thoracic Society/European Respiratory Society guidelines^5,6^ using a Lilly pneumotachograph system with animated incentive software (Jaeger, Germany). For home visits, we used a flow turbine spirometer (Micro Medical, UK). Subjects were asked to inhale to total lung capacity (TLC), then instructed to perform a forced expiration, through a mouthpiece, to residual volume (RV). The test was repeated at intervals of 30 seconds until 3 technically acceptable traces were obtained. Forced expiratory volume in one second (FEV_1_) and forced vital capacity (FVC) were recorded and the data expressed as FEV_1_ % predicted and FEV_1_/FVC ratio. Reference equations from the Global Lung Function Initiative (GLI) were used to calculate percent predicted values according to age, sex, height and ethnicity.

SEATON and Ashford: Pre-bronchodilator lung function tests were conducted at the follow-ups in adolescence. FVC and FEV1 were measured using a Koko Spirometer and software with a portable desktop device (both PDS Instrumentation, Louisville, KY, USA) according to American Thoracic Society/European Respiratory Society guidelines^5,6^.

Study participants were required to be free of respiratory infection for 2 weeks and not to be taking any oral steroids. Short-acting β2-agonists were withheld for at least four, and long-acting for at least 24 hours prior to testing.

**Methacholine challenge**

In MAAS, airway reactivity was assessed through methacholine challenge with a 5-step protocol performed according to American Thoracic Society guidelines. Quadrupling doses of methacholine (0.0625-16.0 mg/mL) were delivered to subjects through a DeVilbiss 646 nebulizer (Sunrise Medical HHG, Somerset, Pa) and a KoKo dosimeter (Pulmonary Data Services, Doylestown, Pa) calibrated to deliver 0.009 mL per 0.6-second actuation. The predicted FEV_1_ was calculated, and if the measured value was less than 1.0 L or less than 60% of the predicted value, the test was not performed. FEV1 was measured 30 and 90 seconds after 5 inhalations of each dose of methacholine. The challenge was stopped when either a 20% decrease in FEV_1_ was observed or the maximum methacholine concentration had been administered.

**FeNO**

In MAAS, FeNO was measured at ages 8, 11, 16 and 18-20 years using either a chemiluminescence analyser (NIOX, Aerocrine, Sweden) or electrochemical analyser (NIOX Mino, Aerocrine, Sweden NIOX), changed on the 4th May 2012. The devices gave comparable results in previous studies. In SEATON, FeNO was ascertained using NIOX chemiluminescence analyser at age 10 years.

**Exacerbations and hospitalisation**

*Severe exacerbation of wheeze/asthma:* Defined from medical records as either receipt of oral corticosteroids (OCS) for at least 3 days, or emergency department visit because of asthma/wheeze requiring systemic corticosteroids, or hospital admission.^7,8^ We ascertained age in days of each exacerbation to provide an accurate account of each episode.

*Lower respiratory tract infection hospital admission:* Extracted form electronic and paper-based medical records.

**Wheeze severity**

“Non wheezers”: Children with no reported episodes of wheezing;

“Mild wheeze”: Children with less than 4 episodes in 12 months, with sleet disturbed less than 1 per week and with no reported limiting speech due to wheezing;

“Moderate/severe wheeze”: Children with more than 4 episodes of wheezing in 12 months, who reported to be woken up more than 1 per week and who experienced limitation to speech due to wheezing.

**Genotyping and imputation**

Genome-wide genotyping platforms

*MAAS:* Study participants were genotyped using the Illumina 610 quad genome-wide SNP genotyping platform (Illumina Inc., San Diego, CA, USA). Prior to imputation samples were excluded on the basis of gender mismatches; minimal or excessive heterozygosity, genotyping call rates of <97%. SNPS were excluded if they had call rates of < 95%, minor allele frequencies of < 0.5% and HWE p<3x10-8. Prior to imputation each chromosome was pre-phased using EAGLE2 (v2.0.5) as recommended by the sanger imputation server^9^. We then imputed with PBWT with the Haplotype Reference Consortium (release 1.1) of 32,470 reference genomes using the Sanger Imputation Server.

*SEATON and ASHFORD:* Participants were genotyped using the Illumina Infinium Omni2.5-8 v1.3 BeadChip genotyping platform (Illumina Inc., San Diego, CA, USA). Genotype QC and imputation was carried out as described for MAAS.

**STATISTICAL ANALYSIS**

*PAM algorithm:* PAM is a model free clustering procedure, similar to popular K-means^10-13^ that relies on the identification of medoids. While K-means identifies k centroids to cluster the observations, PAM selects k central real observations called medoids. A medoid is always a real observation selected from the dataset, instead, a centroid is an artificial point.

The algorithm is formed by two steps: the BUILD and the SWAP step. In the BUILD step k clusters are obtained by selecting k observations working as medoids, allocating the remaining data to the nearest medoid. Then in the SWAP step, the contribution of changing a medoid with a non-medoid is iteratively evaluated. This second step runs until a further switch between a medoid and a non-medoid does not increase the performance of the clustering solution.

Distance measures

The effectiveness of distance-based clustering methods like PAM and K-means relies on the specific distance measure chosen. In this study, we are adopting the simple matching distance for our clustering approach. The simple matching distance is a suitable and interpretable choice for cluster analysis with categorical data due to its applicability, intuitive interpretation, robustness, and computational efficiency.

Here we briefly describe the simple matching distance used to deal with categorical data under a clustering framework.

Given two observations of $j$ binary variables, $y_{l}$ and $y_{m}$ is it possible to obtain a two-dimensional table as the table below, where $o$ is the number of dimensions for which both $y_{l.}$ and $y_{m.}$ assume value 1. Where $y_{l.}$ Represents the value assumed on a single dimension for the observation $l$.

|  | ${y_{l.}=1}$ | ${y_{l.}=0}$ |
| --- | --- | --- |
| ${y_{m.}=1}$ | $o$ | $p$ |
| ${y_{m.}=0}$ | $q$ | $r$ |

The simple matching score is defined as:

|  | $s=\frac{o+r}{o+p+q+r}.$ | 1 |
| --- | --- | --- |

The distance is then obtained as the root square of $1-s$ where $s$is the similarity index.

Performance measures

The definition of a suitable performance measure is a crucial choice to determine the preferable clustering solution. PAM is usually used in combination with silhouette index to determine the most informative data-partition. The index is defined as the mean over all observations j=1…n of:

|  | $\mathrm{sil}\left( j \right)={\frac{e\left( j \right)-i\left( j \right)}{\max\left( e\left( j \right),i\left( j \right) \right)}}^{'}$ | 3 |
| --- | --- | --- |

Here, $i\left( j \right)$ indicates the average distance between observation $i$ and all the other observations in the same cluster, and $e\left( j \right)$ the distance between the observation $i$ and the nearest clusters to which the observation is not assigned.

In the study we decided to rely on the evaluation of 2 additional performance measures: the C-index and the homogeneity.

The $C-index$ id defined as:

|  | $\frac{T_{l}-T_{m\mathrm{in}}}{T_{m\mathrm{ax}}-T_{m\mathrm{in}}}.$ | 4 |
| --- | --- | --- |

$T_{l}$ is obtained summing all $M_{l}$ pairs distances considering the clustering partition, while $T_{m\mathrm{in}}$ and $T_{max}$ are the sum of the $M_{l}$ lowest and highest pairwise-distances in the entire set of data.

Both the silhouette index and the C-index evaluate the performance of a partition based on the distribution of the data used to compute the distance measure. An external performance measure, instead, evaluates the performance of a clustering solution based on the distribution of an external variable, not originally used to cluster the observations. Using this type of measures to cluster the observations, would allow to select the number of cluster k for which the distribution of a variable of interest is more or less homogenously distributed among clusters. We used as measure the homogeneity index described by ^14^. We would prefer a high value of homogeneity as it would be associated with a solution characterized by clusters with the same outcome variable.

Using $K$to indicate the clusters and $C$ the outcome classes, let $a_{ck}$ be the number of observations in cluster $k$ with the outcome $c$, we can define the homogeneity as

|  |  |  |
| --- | --- | --- |
|  | $\text{ }\text{hom}\text{=}\left\{ \begin{aligned} &1 &&\text{ if }H(C,K)=0 \\ &1-\frac{H\left( C \mid K \right)}{H\left( C \right)} &&\text{ otherwise } \end{aligned} \right.$ | 5 |

for which:

|  | $H\left( C \mid K \right)=-\sum_{k=1}^{\left\vert K \right\vert} \sum_{c=1}^{\left\vert C \right\vert} \frac{a_{ck}}{N}\log\frac{a_{ck}}{\sum_{c=1}^{\left\vert C \right\vert} a_{ck}}$ | 6 |
| --- | --- | --- |
|  | $H\left( C \right)=-\sum_{c=1}^{\left\vert C \right\vert} \frac{\sum_{k=1}^{\left\vert K \right\vert} a_{ck}}{n}\log\frac{\sum_{k=1}^{\left\vert K \right\vert} a_{ck}}{n}$ | 7 |
|  |  |  |

and

|  | $H\left( C,K \right)=-\sum_{k=1}^{K} \sum_{c=1}^{C} \frac{a_{ck}}{N}\log\left( \frac{a_{ck}}{\sum_{c=1}^{C} a_{ck}} \right)$ | 8 |
| --- | --- | --- |

***Association of clusters with early−life risk factors and objective outcomes***

The association of clusters with early-life risk factors and objective outcomes was evaluated using appropriate statistical tests based on data characteristics. The Kruskal-Wallis test was applied for comparing continuous variables across multiple clusters to assess median differences, while the Wilcoxon test was used for pairwise comparisons of continuous variables between two groups. For categorical variables, the Fisher exact test was employed when expected frequencies were low, and the Chi-squared test was used for larger sample sizes to test associations between clusters and categorical variables.

To ensure the readability of the text, we have chosen to highlight the differences between clusters in Table 1, rather than displaying pairwise tests (10 pairs), which would be difficult to present clearly. While we described clear differences between specific clusters (clusters 2 and 4) based on the reported symptoms, we have also included the main differences we found to be interesting for distinguishing between the two groups, such as FENO and severity.

**Acknowledgements:** We thank study participants and their parents for their continued support and enthusiasm, and greatly appreciate the commitment they have given to the project. We also acknowledge the hard work and dedication of the study teams (post-doctoral scientists, physiologists, research fellows, nurses, technicians, and clerical staff).

**Table S1.** Questions used in each cohort.

|  | **QUESTIONS** |
| --- | --- |
| M  A  A  S | History of wheeze: “Has your child ever had wheezing or whistling in the chest at any time in the past?” at the latest follow-up (age 5) |
|  | “In the past 12 months, has wheezing been severe enough to limit your child’s speech to only one or two words at a time between breaths?” |
|  | “In the past 12 months has your child’s chest sounded wheezy during or after exercise?” |
|  | “In the past 12 months has your child's chest sounded wheezy when he / she had not recently taken exercise?” |
|  | “In the past 12 months has your child had wheezing or whistling in the chest when he / she had a cold or the flu?” |
|  | “In the past 12 months has your child had wheezing or whistling in the chest when he / she did not have a cold or the flu?” |
|  | “Has your child woken up with shortness of breath at any time in his / her life?” |
|  | “Has your child woken up with tightness of the chest at any time in his / her life?” |
|  | wheeze triggers: weather, pollen, flu, dust, pets, fumes, and emotions. |
| S  E  A  T  O  N | History of wheeze: “Has your child ever had wheezing in the chest any time in the past?” At the last follow up |
|  | Has your child woken up with shortness of breath at any time in his/her life? |
|  | Has your child woken up with tightness of the chest at any time in his/her life? |
|  | In the last 12 months has your child had wheezing in the chest when he / she had a cold or the flu? |
|  | In the last 12 months has your child had wheezing in the chest when he / she did not have a cold or the flu? |
|  | In the last 12 months has the wheezing been severe enough to limit your child’s speech to only one or two words at a time between breaths? |
| A  S  H  F  O R  D | History of wheeze: retrieved by questions at each follow up concerning wheezing in the last 12 months until latest follow-up |
|  | Wheeze with cold positive answer to: wheezing episodes in the last 12 months AND ("He/She Wheezes Only When He/She Has A Cold Or Other Infection" OR "Having A Cold Or Other Infection Makes Him/Her Wheezing Worse") |
|  | Wheeze without cold positive answer to: wheezing episodes in the last 12 months AND ("Having A Cold Or Other Infection Does Not Make Him/Her Wheezing Worse") OR ("Having A Cold Or Other Infection Makes Her Wheezing Worse") |
|  | In the last twelve months has her wheezing been severe enough to limit her speech to only one or two words at a time between breaths? |
|  | Trigger pets: In the last twelve months have any animals or pets made her wheezy? |
|  | Trigger exercise positive answers to: "In the last twelve months has running around or any other exercise ever made her wheezy?" OR "In the last twelve months has going to the swimming pool ever made her wheezy?" |
|  | Trigger dust: In the last twelve months has dust in the home made her wheezy? |
|  | Trigger weather positive answers to: "In the last twelve months have changes in temperature ever made her wheezy?" OR "In the last twelve months has cold or foggy weather ever made her wheezy?" |
|  | Trigger weather positive answers to: "In the last twelve months has being excited ever made her wheezy?" OR In the last twelve months has crying ever made her wheezy? |

**SUPPLEMENTARY RESULTS**

**Table S2.** Demographic characteristics of the discovery population (MAAS); The numbers in parenthesis denote the sample size for each analysis and reflect the availability of data.

| **Variable** | ***Frequency/Percent (n=947)*** |
| --- | --- |
| Asthma diagnosis (age 8) | 17.11% (162/947) |
| Sex (F) | 46.04% (436/947) |
| Mode of delivery (Vaginal) (recruitment,) | 78.54% (622/792) |
| Maternal smoking (Yes) (recruitment,) | 13.04% (123/943) |
| Maternal asthma ever (yes) (recruitment,) | 19.85% (188/947) |
| Maternal current asthma (yes) (recruitment,) | 14.41% (136/944) |
| Maternal hay-fever (yes) (recruitment,) | 27.06% (256/946) |
| Maternal eczma (yes) (recruitment,) | 15.63% (148/947) |
| Paternal smoking (yes) (recruitment,) | 25.19% (238/945) |
| Paternal asthma ever (yes) (recruitment,) | 14.68% (139/947) |
| Paternal current asthma (yes) (recruitment,) | 8.38% (79/943) |
| Paternal hay fever (yes) (recruitment,) | 24.08% (228/947) |
| Paternal eczema (yes) (recruitment,) | 10.67% (101/947) |
| Older sibling with asthma (yes) | 10.3% (97/942) |
| Older sibling with eczema (yes) (recruitment,) | 14.12% (133/942) |
| Older sibling hay-fever (yes) (recruitment,) | 3.50% (33/942) |
| Ever pet owner by age 5 | 30.53% (283/927) |
| *Allergic sensitisation* |  |
| Mite (age 8) | 20.65% (177/857) |
| Cat (age 8) | 12.25% (105/857) |
| Dog (age 8) | 3.15% (27/857) |
| Grass (age 8) | 16.57% (142/857) |
| At least one allergen (age 8) | 41.11% (356/866) |
| *AHR* |  |
| Positive methacholine challenge (age 8) | 17.83% (107/600) |
| *Airway inflammation* |  |
| FeNO: Median (ppb) (age 8) | 9.7 (402/947) |
| *Lung function* |  |
| FEV_1_/FVC, Median (age 8) | 86.84 (738/947) |

**Table S3.** The frequency distribution of the 15 variables used for cluster derivation in MAAS.

| **Variable** | ***Frequency/Percent (n=947)*** |
| --- | --- |
| (1) Wheeze ever to age 5 years | 39.39% (373/947) |
| *Current wheeze triggers (age 8 years)* |  |
| (2) Wheeze with exercise | 7.29% (69/947) |
| (3) Wheeze without exercise | 12.25% (116/947) |
| (4) Wheeze with colds | 14.89% (141/947) |
| (5) Wheeze apart from colds | 8.45% (80/947) |
| (6) Wheeze with changes in weather | 7.92% (75/947) |
| (7) Wheeze with exposure to pollen | 4.86% (46/947) |
| (8) Wheeze with flu | 14.26% (135/947) |
| (9) Wheeze with exposure to dust | 2.96% (28/947) |
| (10) Wheeze with exposure to pets | 3.59% (34/947) |
| (11) Wheeze with exposure to fumes | 1.59% (15/947) |
| (12) Wheeze triggered by emotions | 2.32% (22/947) |
| *Indicators of wheeze severity (age 8 years)* |  |
| (13) Wheeze limiting speech | 2.85% (27/947) |
| *Other symptoms (age 8 years)* |  |
| (14) Shortness of breath | 16.37% (155/947) |
| (15) Tightness of the chest | 18.16% (172/947) |

**Figure S1. Evaluation of performance measures for different number of clusters for MAAS cohort**

The three lines represent the solution derived using 3 different random initialisations. For each number of clusters, the black line represents the mean value of the performance measure obtained in the three repetitions. The grey lines represent respectively the minimum and the maximum value obtained in the three repetitions for a given number of clusters.


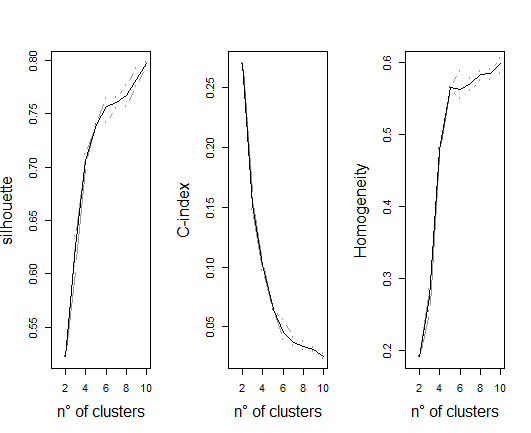
Increasing the number of clusters would reduce the cluster size and would not affect the homogeneity index**.**

**Table S4:** Distribution of non-significant risk factors among retrieved clusters based on Fisher exact test.

|  | **Cluster 1**  532/947 (56.18%) | **Cluster 2**  73/947 (7.71%) | **Cluster 3**  70/947 (7.39%) | **Cluster 4**  64/947 (6.76%) | **Cluster 5**  208/947 (21.96%) |
| --- | --- | --- | --- | --- | --- |
| ***Demographic/early life characteristics*** |  |  |  |  |  |
| Mode of delivery (Vaginal) | 78.62%  320/407 | 75.36%  52/69 | 77.78%  49/63 | 74.58%  44/59 | 80.93%  157/194 |
| Maternal smoking (Yes) | 11.28%  60/532 | 14.29%  10/70 | 14.29%  10/70 | 14.06%  9/64 | 16.43%  34/207 |
| Maternal hay-fever (yes) | 26.32%  140/532 | 27.40%  20/73 | 18.57%  13/70 | 31.75%  20/63 | 30.29%  63/208 |
| Maternal eczema (yes) | 14.10%  75/532 | 19.18%  14/73 | 20.00%  14/70 | 17.19%  11/74 | 16.35%  34/208 |
| Paternal smoking (yes) | 22.56%  120/532 | 24.66%  18/72 | 27.14%  19/70 | 35.94%  23/64 | 27.88%  58/208 |
| Paternal asthma ever (yes) | 13.35%  71/532 | 21.92%  16/73 | 10.00%  7/70 | 20.31%  13/64 | 15.38%  32/208 |
| Paternal current asthma (yes) | 7.33%  39/532 | 11.43%  8/70 | 8.57%  6/70 | 9.38%  6/64 | 9.66%  20/207 |
| Paternal hay fever (yes) | 24.62%  131/532 | 30.14%  22/73 | 15.71%  11/70 | 21.88%  14/64 | 24.04%  50/208 |
| Paternal eczema (yes) | 10.71%  57/532 | 12.33%  9/73 | 8.57%  6/70 | 10.94%  7/64 | 10.58%  22/208 |
| Older sibling with eczema (yes) | 13.16%  70/532 | 21.74%  15/69 | 21.43%  15/70 | 12.50%  8/64 | 12.08%  25/207 |
| Older sibling hay-fever (yes) | 2.82%  15/532 | 7.24%  5/69 | 4.29%  3/70 | 6.25%  4/64 | 2.9%  6/207 |
| Ever pet owner by age 5 | 30.23%  159/526 | 37.14%  26/70 | 26.15%  17/65 | 30.65%  19/62 | 30.39%  62/204 |

Figure S2. Airway inflammation and lung function in the 5 clusters in the MAAS cohort: cross-sectional analysis. The solid line represents the median level for each cluster. Circular points indicate children without asthma diagnosis, while crosses indicate children with asthma diagnosis. Differences were assessed using Kruskal-Wallis test

1. FeNO (ppb): p-value<0.001 – effect size through correlation ratio: 0.46
2. FEV_1_/FVC (%): p-value<0.001 – effect size through correlation ratio: 0.26

**
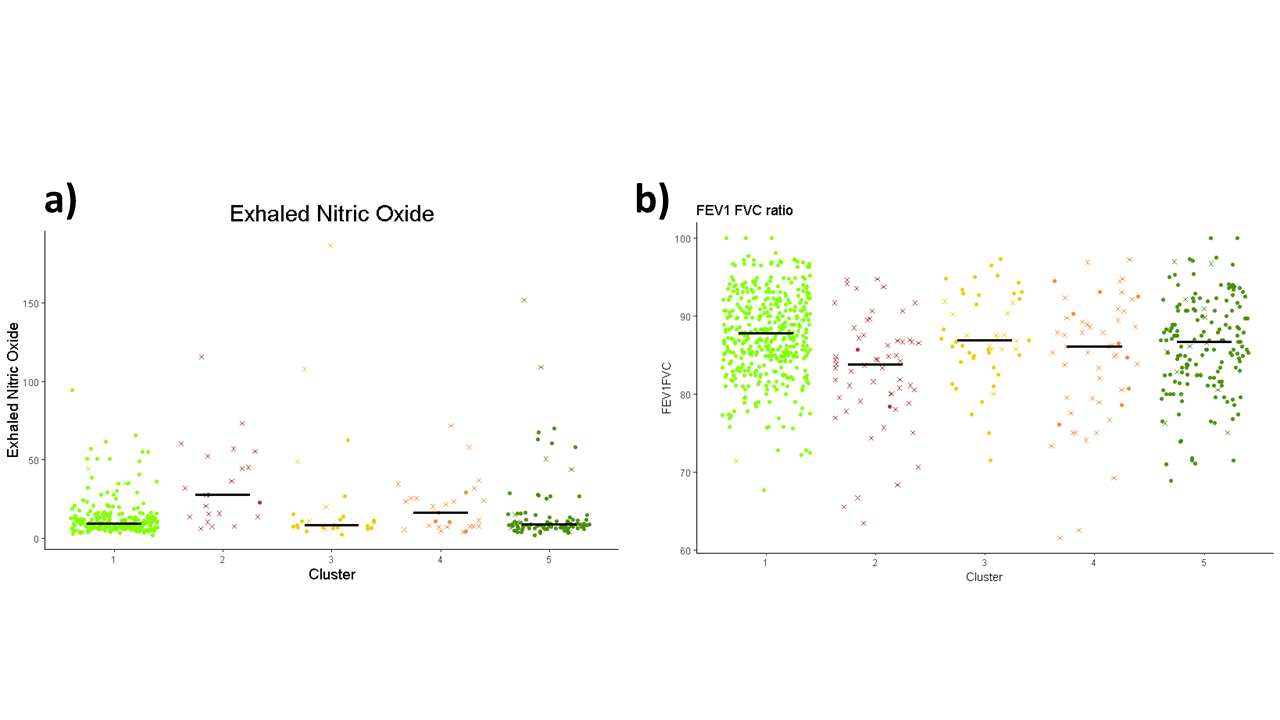
a)**

**b)**

**
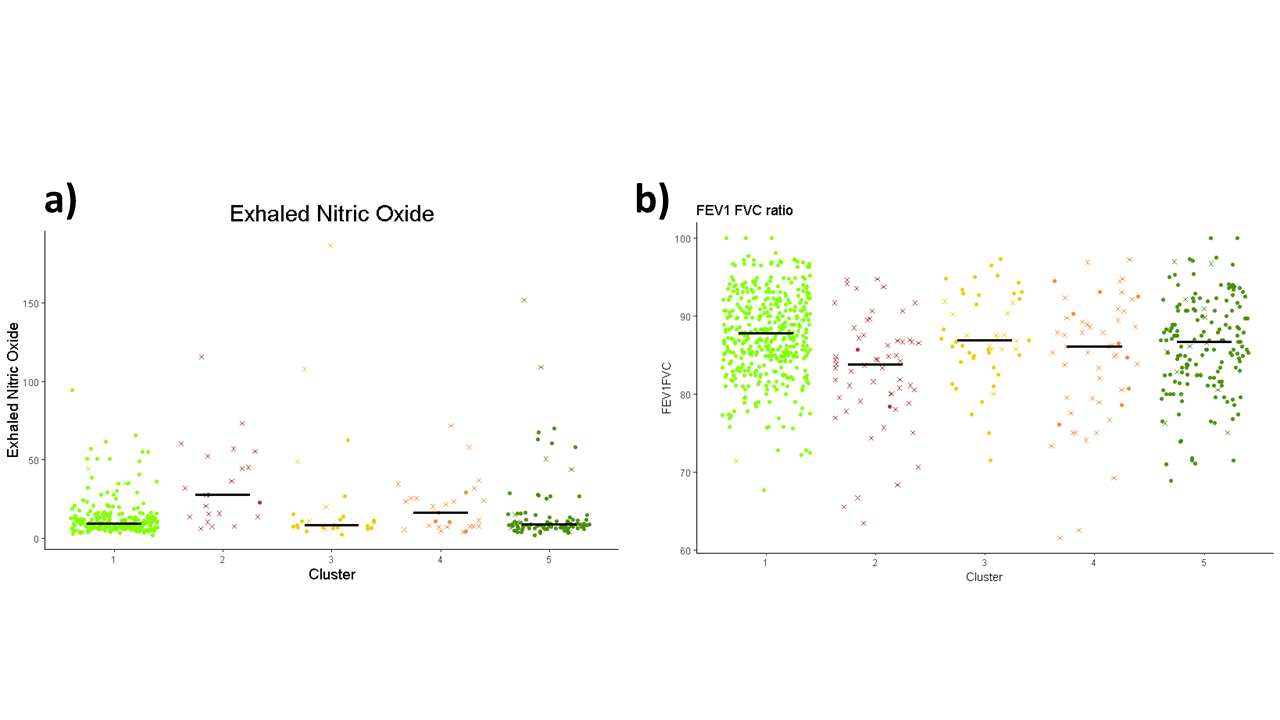
**

**Figure S3.** Median level of FeNo (a) and FEV_1_/FVC (b) over time across clusters.

The legend above the graph reports the number of observations used to compute the median level at each time point (age 8, 11, 16 and 18-20 years) for each cluster.

a) FeNO

**
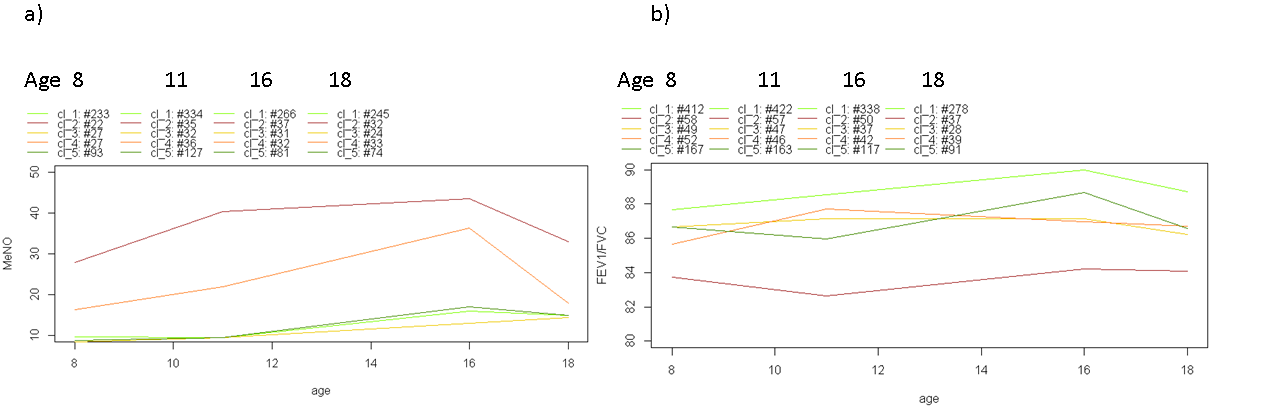
** **Age:** 8 years 11 years 16 years 18-20 years

b) FEV_1_/FVC

**
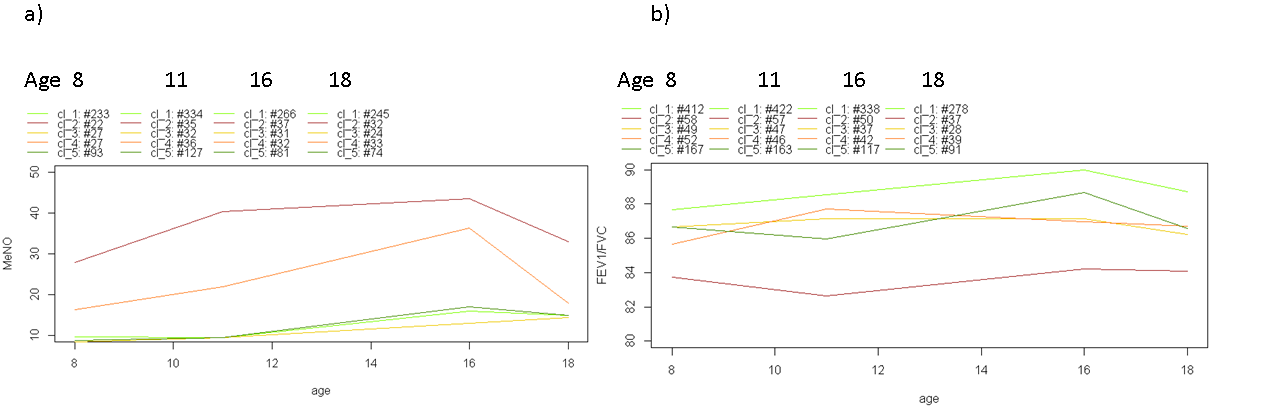
** **Age:** 8 years 11 years 16 years 18-20 years

**Figure S4.** Percentages of sensitisations over time across clusters.

The table below the graphs reports the number of observations used to compute the median level at each time point (age 1, 3, 5, 8, 11, 16 and 18-20) for each cluster. Sample size at each time point is displayed below graphs.


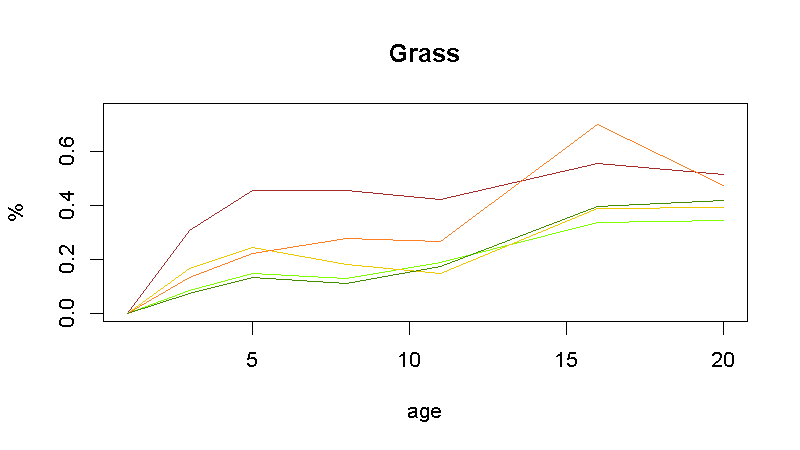
a) grass pollen sensitization

|  | Age 1 | Age 3 | Age 5 | Age 8 | Age 11 | Age 16 | Age 20 |
| --- | --- | --- | --- | --- | --- | --- | --- |
| cl 1 | 250 | 469 | 483 | 470 | 422 | 337 | 280 |
| cl2 | 25 | 52 | 68 | 68 | 57 | 47 | 39 |
| cl3 | 17 | 60 | 66 | 61 | 47 | 36 | 28 |
| cl4 | 27 | 52 | 59 | 61 | 45 | 43 | 38 |
| cl5 | 87 | 177 | 189 | 197 | 163 | 116 | 91 |


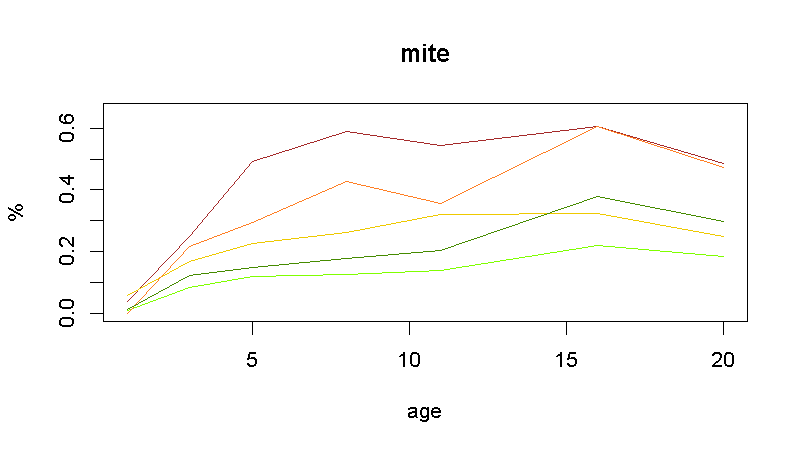
b) House dust mite

|  | Age 1 | Age 3 | Age 5 | Age 8 | Age 11 | Age 16 | Age 20 |
| --- | --- | --- | --- | --- | --- | --- | --- |
| cl 1 | 250 | 469 | 482 | 470 | 422 | 338 | 280 |
| cl2 | 25 | 52 | 69 | 68 | 57 | 48 | 39 |
| cl3 | 17 | 59 | 66 | 61 | 47 | 37 | 28 |
| cl4 | 27 | 51 | 58 | 61 | 45 | 43 | 38 |
| cl5 | 87 | 177 | 189 | 197 | 163 | 116 | 91 |

c) Dog
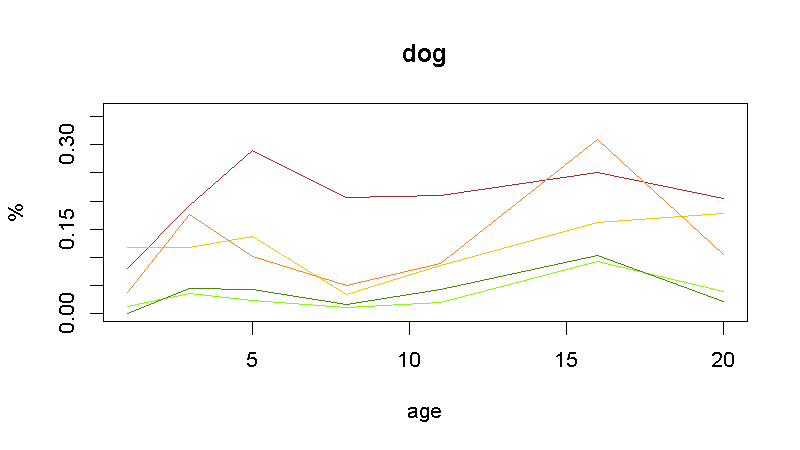


|  | Age 1 | Age 3 | Age 5 | Age 8 | Age 11 | Age 16 | Age 20 |
| --- | --- | --- | --- | --- | --- | --- | --- |
| cl 1 | 250 | 470 | 483 | 470 | 422 | 337 | 280 |
| cl2 | 25 | 52 | 69 | 68 | 57 | 48 | 39 |
| cl3 | 17 | 60 | 66 | 61 | 47 | 37 | 28 |
| cl4 | 27 | 51 | 59 | 61 | 45 | 42 | 38 |
| cl5 | 87 | 177 | 189 | 197 | 163 | 117 | 91 |

d) Cat


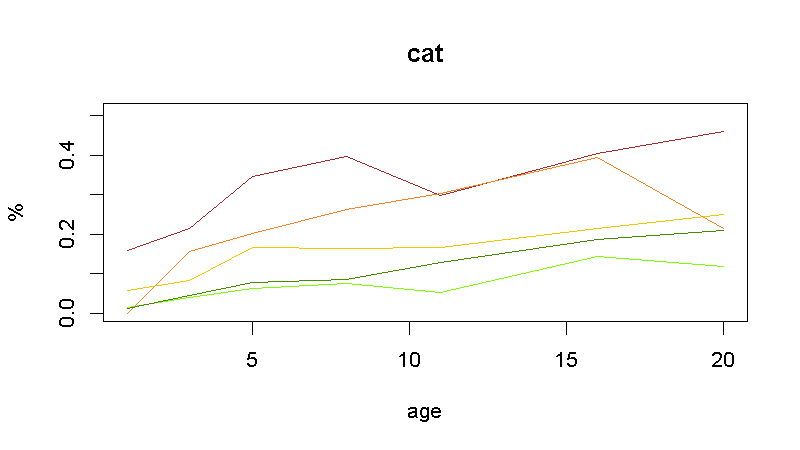


|  | Age 1 | Age 3 | Age 5 | Age 8 | Age 11 | Age 16 | Age 20 |
| --- | --- | --- | --- | --- | --- | --- | --- |
| cl 1 | 250 | 468 | 482 | 470 | 422 | 336 | 280 |
| cl2 | 25 | 51 | 69 | 68 | 57 | 47 | 39 |
| cl3 | 17 | 60 | 66 | 61 | 48 | 37 | 28 |
| cl4 | 27 | 51 | 59 | 61 | 46 | 43 | 37 |
| cl5 | 87 | 176 | 189 | 197 | 164 | 117 | 91 |

**Figure S5.** Percentages of positive methacholine challenge over time across clusters.

The legend above the graph reports the number of observations used to compute the median level at each time point (age 8, 11, and 18-20) for each cluster.

**
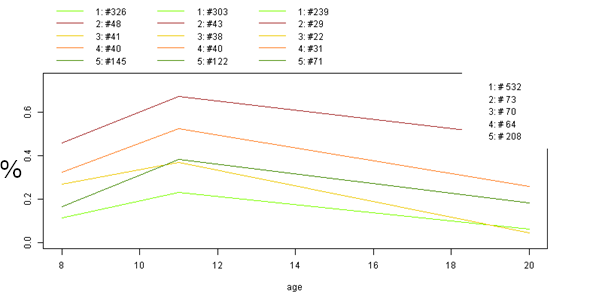
** **Age:** 8 years 11 years 16 years 18-20 years

**VALIDATION**

**SEATON**

Due to differences in the questionnaire administered compared to MAAS, we used overlapping questions representing 6 different variables (Table S2): wheeze ever, wheeze with cold, wheeze without cold, speech limited by wheeze, ever woken with shortness of breath and ever woken with tightness in the chest. As the questionnaires were differently structured, some inconsistency may occur (e.g. some parents can respond negatively to the questions “Has your child had wheezing in the chest in the last 12 months (but not from the throat or nose)?)”, and despite this report wheezing with or without cold during the same period of time. This is not possible following MAAS structure. Here we considered the answer to “Has your child had wheezing in the chest in the last 12 months (but not from the throat or nose)?” correct and predominant and consequently, in case of a negative response, all the reported wheezing symptoms during the last 12 months were considered as negative. The results using the original data, as well as another reasonable correction are presented.

Even without the complete information available in MAAS, the evaluation of the performance measures identified 5-cluster solution as being optimal (Figure S3).

**Figure S6.** Evaluation of performance measures for different number of clusters for SEATON cohort

The three lines represent the solution derived using 3 different random initialisations. For each number of clusters, the black line represents the mean value of the performance measure obtained in the three repetitions. The grey lines represent respectively the minimum and the maximum value obtained in the three repetitions for a given number of clusters


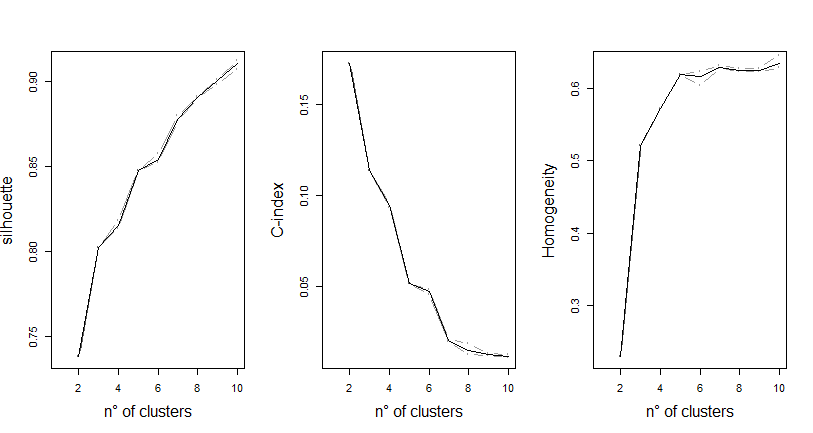


**Figure S7. Distribution of variables within the 5 clusters retrieved in the SEATON cohort**

The distribution of the variables used to create the clusters are compared with the distribution of the same variables in all the data set, depicted in grey. The position of each dot represents the percentage of children with positive answer to the associated question for a specific cluster. Each dot is positioned on an axis with range limit 0% and 100%.


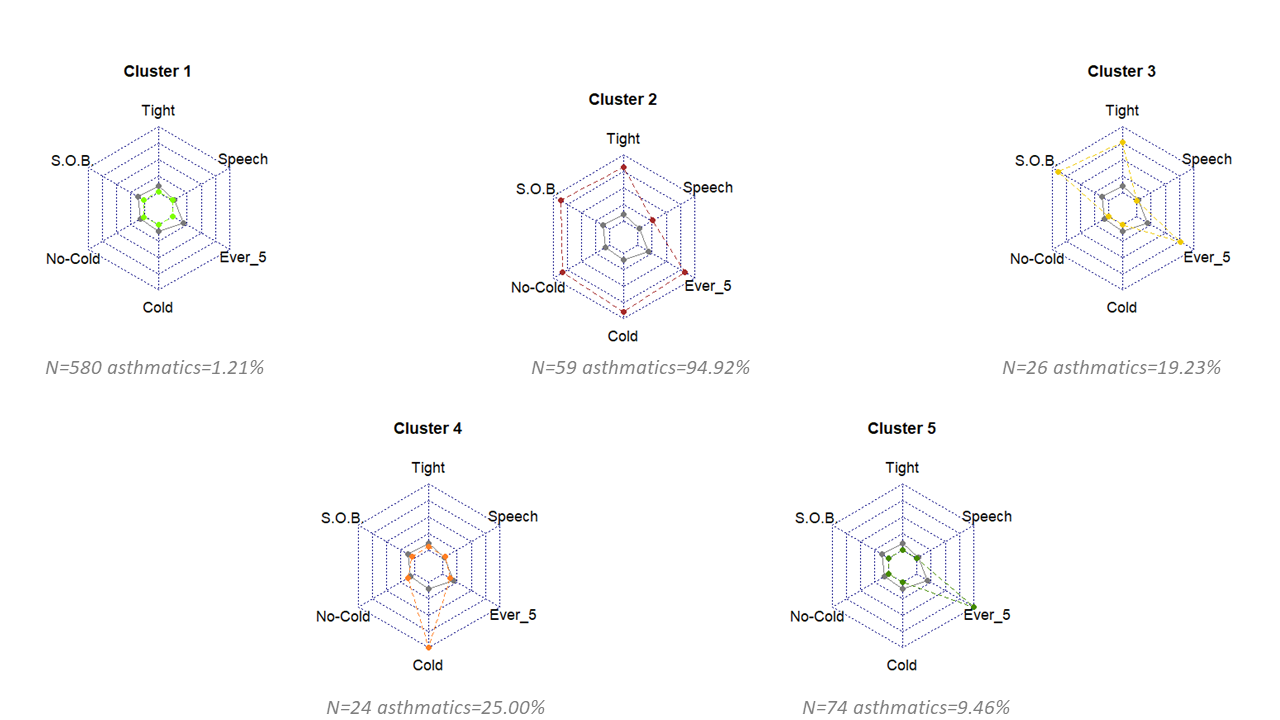


**Table S5:** SEATON - Distribution of the variables used to derive the clusters and risk factors distribution among the retrieved clusters.

|  | **Cluster 1**  580/763 (76.02%) | **Cluster 2**  59/763 (7.73%) | **Cluster 3**  26/763 (3.41%) | **Cluster 4**  24/763 (3.15%) | **Cluster 5**  74/763 (9.70%) |
| --- | --- | --- | --- | --- | --- |
| Wheeze ever to age 5 years | 0.00% | 83.05% | 76.92% | 12.50% | 100.00% |
| ***Wheeze triggers*** |  |  |  |  |  |
| Wheeze with colds | 0.00% | 89.83% | 0.00% | 100.00% | 0.00% |
| Wheeze apart from colds | 0.86% | 83.05% | 0.00% | 12.50% | 0.00% |
| ***Wheeze severity (age 10)*** |  |  |  |  |  |
| Wheeze limiting speech | 0.17% | 25.42% | 0.00% | 4.17% | 0.00% |
| ***Other symptoms (age 10 years)*** |  |  |  |  |  |
| Shortness of breath | 2.24% | 86.44% | 88.46% | 4.17% | 0.00% |
| Tightness in the chest | 0.52% | 81.36% | 76.92% | 4.17% | 0.00% |
| ***Demographic/early life characteristics*** |  |  |  |  |  |
| Maternal smoking (Yes) (recruitment) | 17.76%  103/580 | 20.34%  12/59 | 19.23%  5/26 | 12.5%  3/24 | 27.03%  20/74 |
| Maternal asthma ever (yes) (recruitment) | 11.72%  68/580 | 22.03%  13/59 | 19.23%  5/26 | 25%  6/24 | 21.62%  16/74 |
| Maternal current asthma (yes) (recruitment) | 7.24%  42/580 | 15.25%  9/59 | 11.54%  3/26 | 16.67%  4/24 | 16.22%  12/74 |
| Maternal hay-fever (yes) (recruitment) | 21.55%  125/580 | 44.07%  26/59 | 30.77%  8/26 | 37.5%  9/24 | 28.38%  21/74 |
| Maternal eczema (yes) (recruitment) | 15%  87/580 | 32.2%  19/59 | 23.08%  6/26 | 20.83%  5/24 | 22.97%  17/74 |
| Ever pet owner by age 5 | 54.18%  311/574 | 65.52%  38/58 | 50%  13/26 | 60.87%  14/23 | 54.05%  40/74 |
| ***Wheeze severity (age 10 years)*** |  |  |  |  |  |
| Number of wheezing episodes: 0 | 99.14% | 0.00% | 100.00% | 0.00% | 100.00% |
| Number of wheezing episodes: [1-3] | 0.86% | 37.29% | 0.00% | 87.50% | 0.00% |
| Number of wheezing episodes: [4-12] | 0.00% | 45.76% | 0.00% | 8.33% | 0.00% |
| Number of wheezing episodes: >12 | 0.00% | 11.86% | 0.00% | 4.17% | 0.00% |
| Don't know | 0.00% | 5.08% | 0.00% | 0.00% | 0.00% |
| ***Lung function/Airway inflammation (age 10 years)***  Median and IQR |  |  |  |  |  |
| FeNO | 9.30  IQR=7.10 | 37.00  IQR=44.38 | 7.00  IQR=6.40 | 25.8  IQR=34.85 | 8.40  IQR=3.80 |
| FEV_1_/FVC | 88.31%  IQR=6.33% | 87.10%  IQR=9.03% | 83.33% IQR=7.21% | 84.43% IQR=7.09% | 86.96%  IQR=9.07% |
| ***Sensitisations (age 10 years)*** |  |  |  |  |  |
| *Cat*  *sensitisation* | 9.82%  28/285 | 42.42%  14/33 | 25%  3/12 | 33.33%  4/12 | 6.45%  2/31 |
| Dog  *sensitisation* | 5.96%  17/285 | 48.48%  16/33 | 8.33%  1/12 | 16.67%  2/12 | 3.23%  1/31 |
| Mite  *sensitisation* | 14.39%  41/285 | 60.61%  20/33 | 25%  3/12 | 33.33%  4/12 | 6.45%  2/31 |
| Grass  *sensitisation* | 20.35%  58/285 | 60.61%  20/33 | 33.33%  4/12 | 41.67%  5/12 | 19.35%  6/31 |

**Figure S8. Distribution of clinical variables within the 5 clusters retrieved in the Seaton cohort**

1. Distribution of FeNO test results are compared among clusters (p-value<0.01; Kruskal-Wallis). The solid line represents the median level for each group.
2. b) Percentages of children sensitised to 4 common allergens divided by cluster membership (p-value<0.001 Fisher test for each of the 4 allergens). Sensitisation was derived by skin prick test using 3mm as threshold

**a) FeNO b) Allergic sensitization**

**
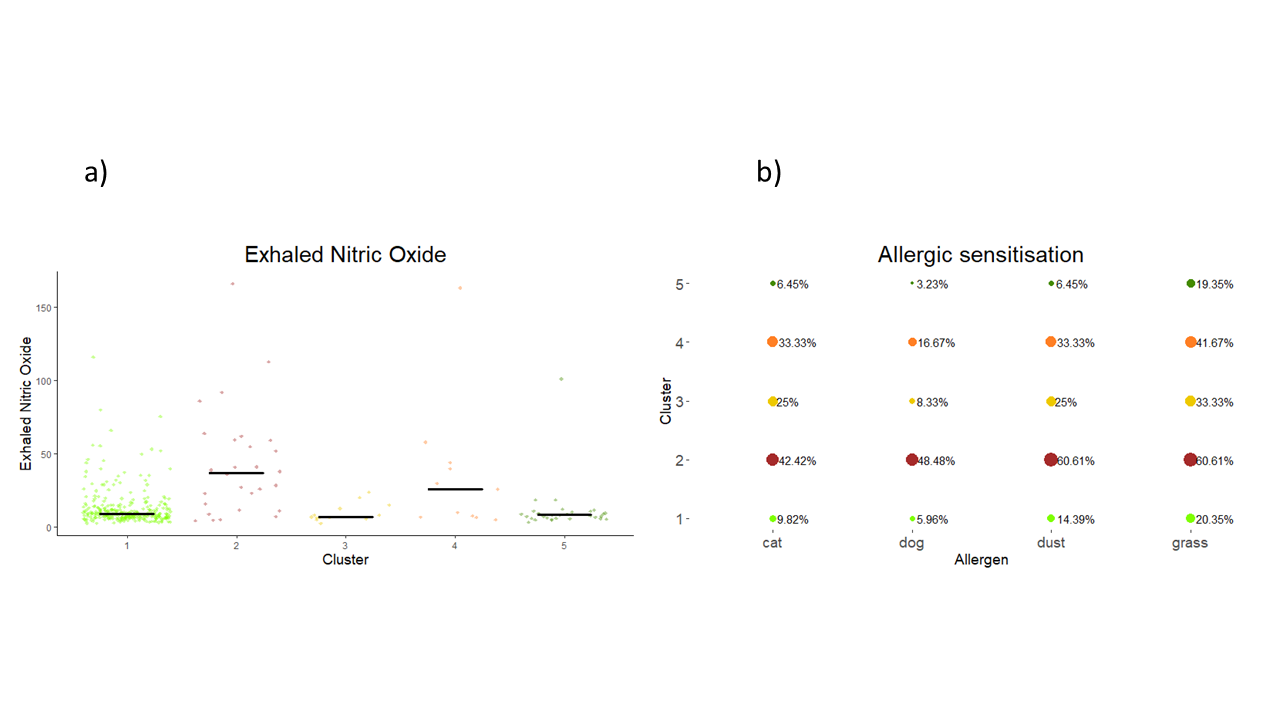
**

SEATON: ALTERNATIVE CHOICES 1

To increase the comparability between MAAS and SEATON questionnaires, we have considered as predominant the answer to the question “Has your child had wheezing in the chest in the last 12 months (but not from the throat or nose)?”. A negative answer to this question has been considered sufficient to ‘skip’ the other questions and receive a negative answer to all the wheezing related questions in the last 12 months, as it is in MAAS.

As other choices could be reasonable, here we present the results using the original data as well as using alternative approaches. In this latter case, when the parents did not know or answered negatively to “Has your child had wheezing in the chest in the last 12 months (but not from the throat or nose)?” we investigated the other wheezing related questions. If any other wheezing related symptoms were reported in the last 12 month, we considered the child as having a wheezing episode in that period.

Analysis among children with complete data

Of the 744 children with complete data, 80 were considered as asthmatics. A 5 clusters solution is suggested (Figure S9) and described in Figure S8. While around 99% and 93% of children in cluster 1 and 5 respectively, did not have any attack of wheezing in the last 12 months, the same percentage for cluster 3 was around 56%. In this cluster around 31% reported from 1 to 3 episodes, 6.25% between 4 and 12 and around 3% more than 12. The same figures for cluster number 2 were: around 35% (1-3 episodes), around 46% (4-12 episodes) and around 13% (>12 episodes). Cluster number 4: around 86% (1-3 episodes), around 7% (4-12 episodes) and around 3% (>12 episodes).

**Figure S9. Evaluation of performance measures for different number of clusters for SEATON cohort using original data. A 5 groups solution is suggested.**


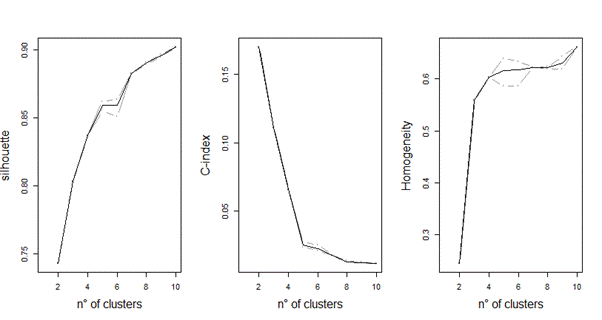
The three lines represent the solution derived using 3 different random initialisations. For each number of clusters, the black line represents the mean value of the performance measure obtained in the three repetitions. The grey lines represent respectively the minimum and the maximum value obtained in the three repetitions for a given number of clusters

**Figure S10. Distribution of variables within the 5 clusters retrieved in the SEATON cohort using complete data.**

The distribution of the variables used to create the clusters are compared with the distribution of the same variables in all the data set, depicted in grey. The position of each dot represents the percentage of children with positive answer to the associated question for a specific cluster. Each dot is positioned on an axis with range limit 0% and 100%.


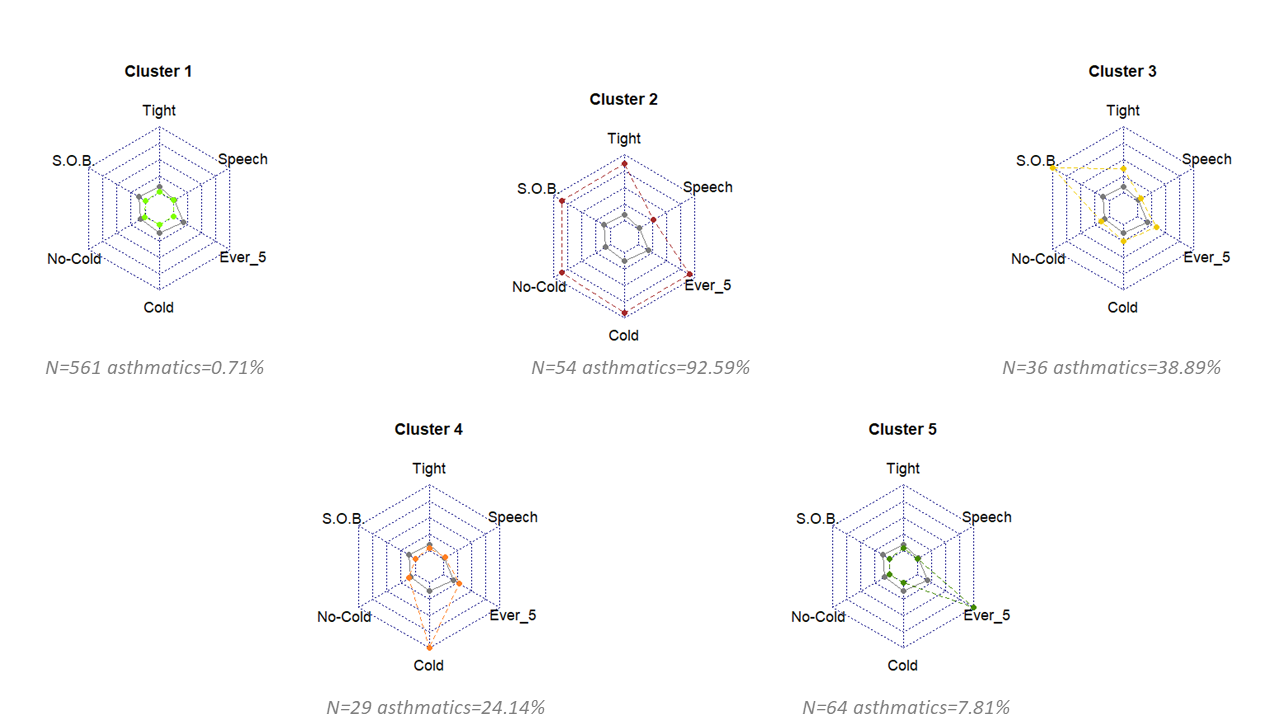


SEATON: ALTERNATIVE CHOICE 2

When the parents did not know or answered negatively to “Has your child had wheezing in the chest in the last 12 months (but not from the throat or nose)?” we investigated the other wheezing related questions. If any other wheezing related symptoms were reported in the last 12 month, we considered the child as having a wheezing episode in that period.

Of the 744 children with complete data, 96 were considered as asthmatics. A 5 clusters solution is suggested (Figure S11) and described in Figure S9. While around 99% and 95% of children in cluster 1 and 5 respectively, did not have any attack of wheezing in the last 12 months, the same percentage for cluster 3 was around 55%. In this cluster around 33% reported from 1 to 3 episodes, 6% between 4 and 12 and around 3% more than 12. The same figures for cluster number 2 were: around 35% (1-3 episodes), around 45% (4-12 episodes) and around 13% (>12 episodes). Cluster number 4: around 86% (1-3 episodes), around 7% (4-12 episodes) and around 3% (>12 episodes).

**Figure S11. Evaluation of performance measures for different number of clusters for SEATON cohort using alternative approach. A 5 groups solution is suggested.**


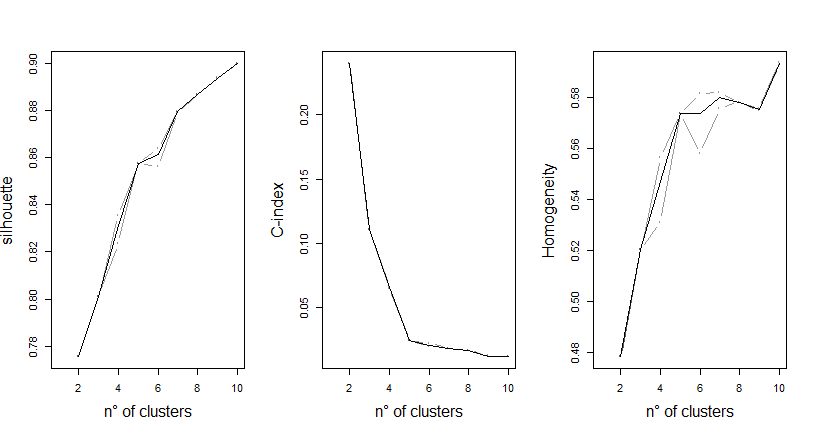
The three lines represent the solution derived using 3 different random initialisations. For each number of clusters, the black line represents the mean value of the performance measure obtained in the three repetitions. The grey lines represent respectively the minimum and the maximum value obtained in the three repetitions for a given number of clusters

**Figure S12. Distribution of variables within the 5 clusters retrieved in the SEATON cohort using alternative approach**

The distribution of the variables used to create the clusters are compared with the distribution of the same variables in all the data set, depicted in grey. The position of each dot represents the percentage of children with positive answer to the associated question for a specific cluster. Each dot is positioned on an axis with range limit 0% and 100%.


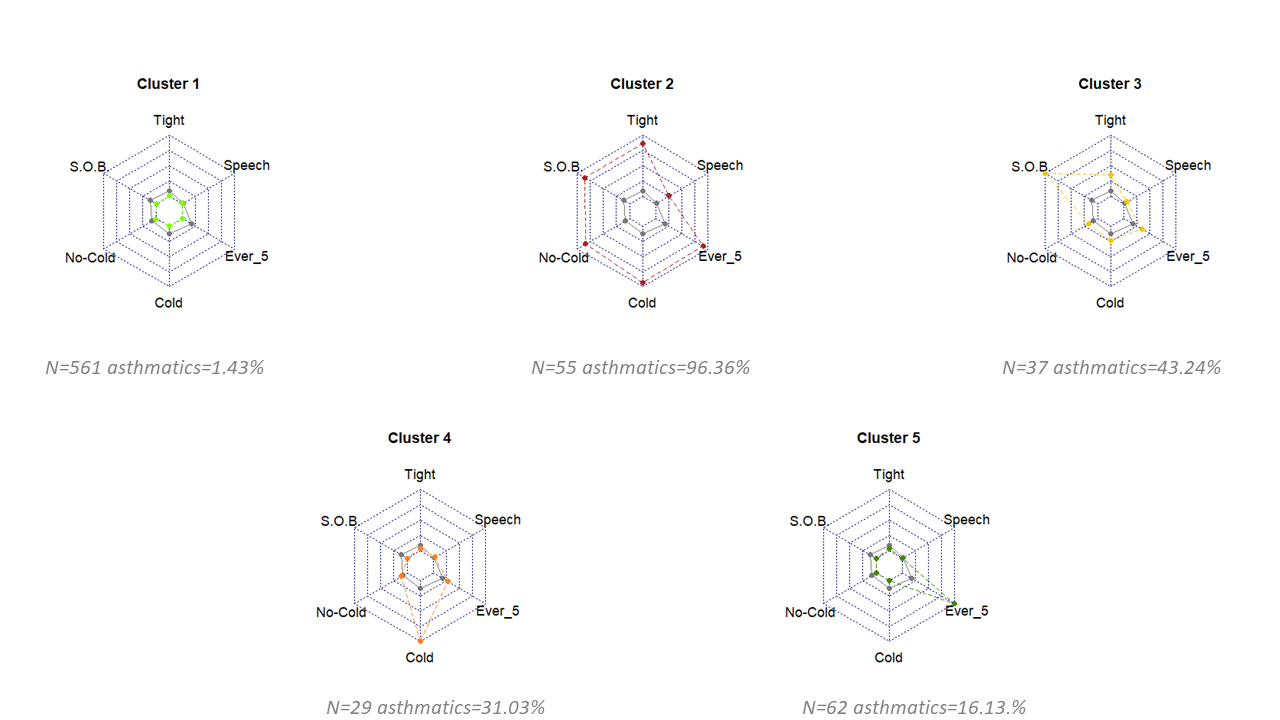


**VALIDATION**

**ASHFORD**

**Figure S13. Evaluation of performance measures for different number of clusters for ASHFORD cohort**

**
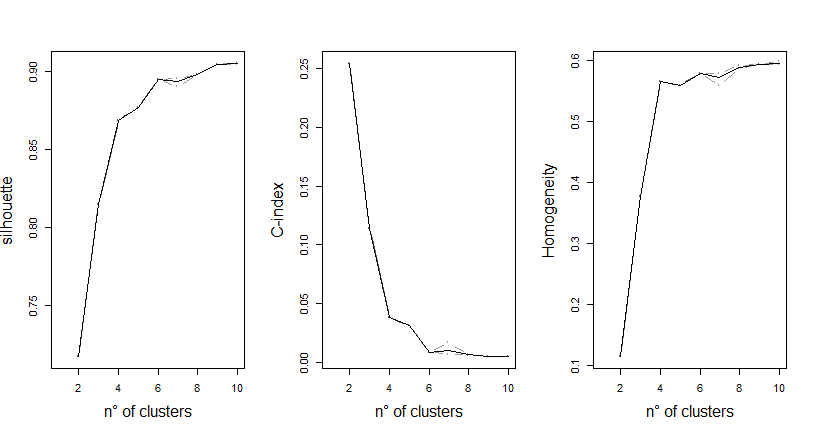
**The three lines represent the solution derived using 3 different random initialisations. For each number of clusters, the black line represents the mean value of the performance measure obtained in the three repetitions. The grey lines represent respectively the minimum and the maximum value obtained in the three repetitions for a given number of clusters.

**Figure S14. Distribution of variables within the 4 clusters retrieved in the Ashford cohort**

The distribution of the variables used to create the clusters are compared with the distribution of the same variables in all the data set, depicted in grey. The position of each dot represents the percentage of children with positive answer to the associated question for a specific cluster. Each dot is positioned on an axis with range limit 0% and 100%.

**
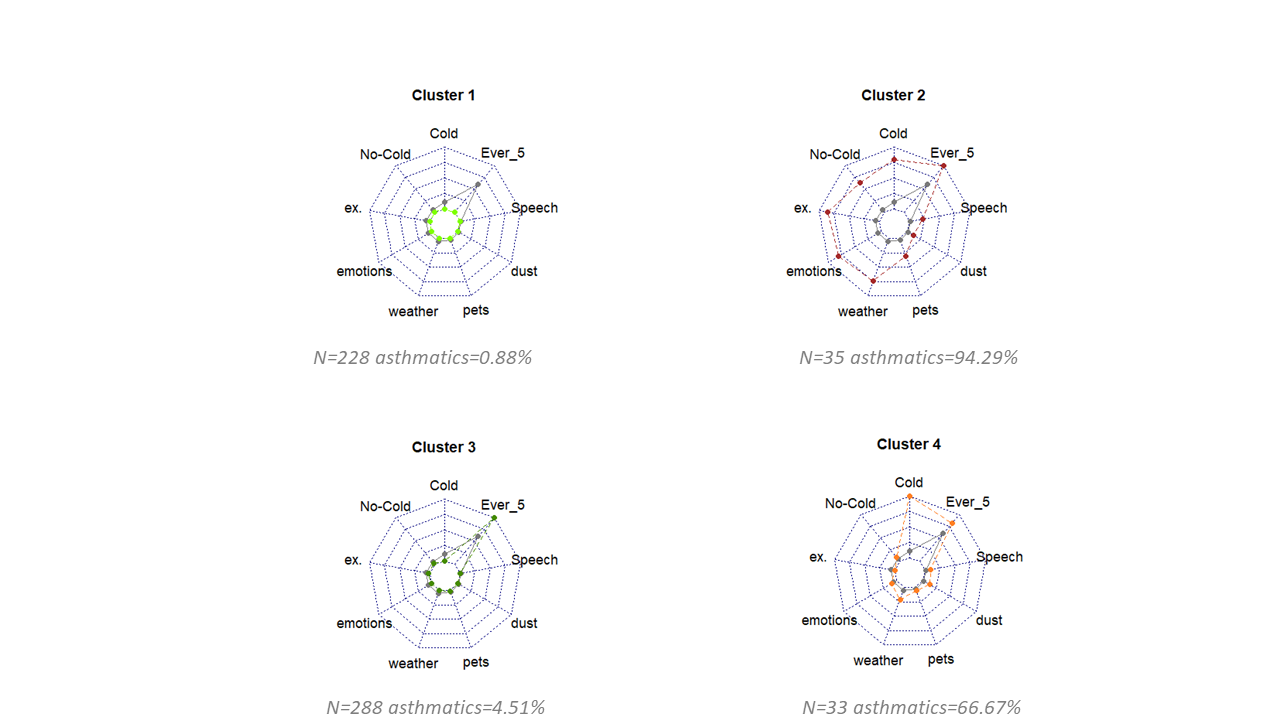
**

**Table S6**: ASHFORD – Distribution of the variables used to derive the clusters and risk factors distribution among the retrieved clusters.

|  | **Cluster 1**  228 /584  (39.04%) | **Cluster 2**  35/584  (5.99%) | **Cluster 3**  288/584  (49.32%) | **Cluster 4**  33/584  (5.65%) |
| --- | --- | --- | --- | --- |
| Wheeze ever to age 5 years | 0.00% | 100.00% | 100.00% | 81.82% |
| ***Wheeze triggers (age 8)*** |  |  |  |  |
| Wheeze with exercise | 0.00% | 85.71% | 1.74% | 0.00% |
| Wheeze with colds | 0.00% | 80.00% | 0.00% | 100.00% |
| Wheeze apart from colds | 0.44% | 62.86% | 2.78% | 9.09% |
| Wheeze with changes in weather | 0.00% | 74.29% | 0.35% | 21.21% |
| Wheeze with exposure to dust | 0.00% | 11.43% | 0.00% | 12.12% |
| Wheeze with exposure to pets | 0.00% | 31.43% | 0.69% | 6.06% |
| Wheeze triggered by emotions | 0.00% | 80.00% | 0.00% | 9.09% |
| ***Wheeze severity (age 8)*** |  |  |  |  |
| Wheeze limiting speech | 0.00% | 22.86% | 0.35% | 9.09% |
| ***Demographic/early life characteristics*** |  |  |  |  |
| Maternal current smoking (Yes) (recruitment) | 9.69% 22/227 | 34.29% 12/35 | 21.95% 63/287 | 18.18% 6/33 |
| Maternal asthma ever (yes) (recruitment) | 5.7% 13/228 | 20% 7/35 | 16.67% 48/288 | 33.33% 11/33 |
| Maternal hay-fever ever (yes) (recruitment) | 20.18% 46/228 | 31.43% 11/35 | 28.82% 83/288 | 36.36% 12/33 |
| Maternal eczema ever (yes) (recruitment) | 20.18% 46/228 | 20%  7/35 | 15.97% 46/288 | 24.24% 8/33 |
| Paternal asthma ever (yes) (recruitment) | 11.5% 26/226 | 26.47% 9/34 | 12.2% 35/287 | 30.3% 10/33 |
| Paternal hay fever (yes) (recruitment) | 26.55% 60/226 | 35.29% 12/34 | 22.73% 65/286 | 36.36% 12/33 |
| Paternal eczema ever (yes) (recruitment) | 16.81% 38/226 | 14.29% 5/35 | 12.54% 36/287 | 6.06% 2/33 |
| ***Sensitisations (age 8)*** |  |  |  |  |
| Cat | 5.58% 12/215 | 36.36% 12/33 | 6.82% 18/264 | 24.14% 7/29 |
| Mite | 11.63% 25/215 | 36.36% 12/33 | 7.95% 21/264 | 48.28% 14/29 |

**ANALYSIS IN MAAS, USING ONLY INFORMATION AVAILABLE IN ASHFORD**

**Figure S15. Evaluation of performance measures for different number of clusters for MAAS cohort using a restricted set of variables**

The three lines represent the solution derived using 3 different random initialisations. For each number of clusters, the black line represents the mean value of the performance measure obtained in the three repetitions. The grey lines represent respectively the minimum and the maximum value obtained in the three repetitions for a given number of clusters

**
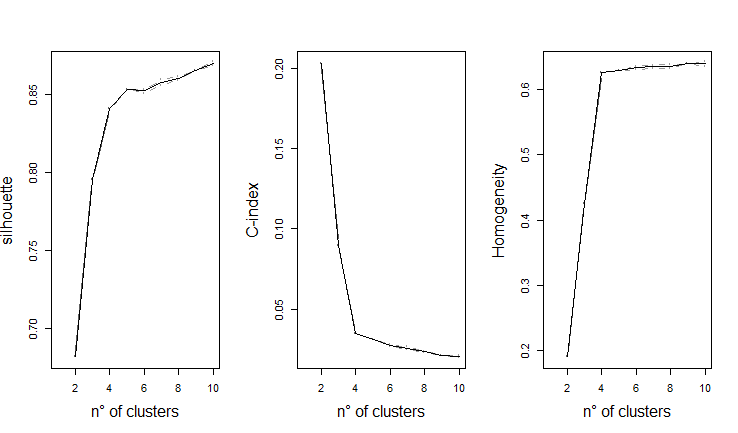
**

**Figure S16. Distribution of variables within the 4 clusters retrieved in the MAAS cohort.**


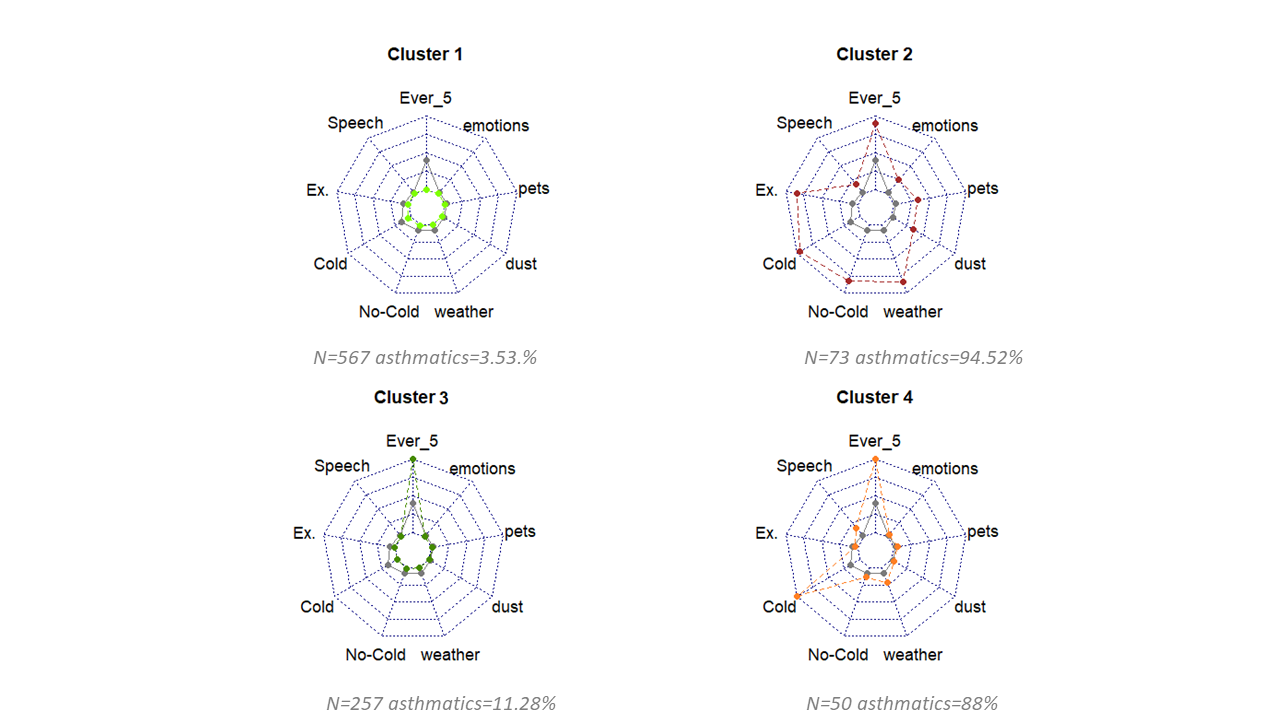
The distribution of the variables used to create the clusters are compared with the distribution of the same variables in all the data set, depicted in grey. The position of each dot represents the percentage of children with positive answer to the associated question for a specific cluster. Each dot is positioned on an axis with range limit 0% and 100%.

**Feature Importance in Deriving Cluster**

We further analyse the significant role played by individual variables in clustering identification (Table S7). Specifically, we compared the Normalised Mutual Index (NMI) obtained by comparing the clustering solution with the full sets of variables with the clustering solution obtained by removing individual features and fixing the number of clusters to 5. This measure offers a quantification of the agreement between two clustering solutions: a value equal to 1 indicates perfect agreement. Moreover, we also provide the NMI by comparing the retrieved clustering solution using the full dataset, with the binary partition offered by individual variables.

Variables including speech limitation, and triggers including pollen, dust, pet, fume, and emotion were individually less important in the clustering identification, with high NMI when individually discarded and low NMI when considered alone.

History of pre-school wheezing, current wheeze with or apart from colds, and current shortness of breath and chest tightness represented the most important symptoms to derive the described clusters.

Based on these variables, clusters can be characterised as:

Cluster 1: No pre-school wheezing and no other symptoms

Cluster 2: History of pre-school wheezing, current wheeze with and apart from colds, shortness of breath and chest tightness

Cluster 3: History of pre-school wheezing, no current wheeze, but current shortness of breath and chest tightness

Cluster 4: History of pre-school wheezing, current wheeze only triggered by cold/flu, no current shortness of breath and chest tightness

Cluster 5: History of pre-school wheezing, but no current wheeze, shortness of breath and chest tightness or any other symptoms.

**Table S7**: Feature importance by Normalized Mutual Information (NMI) in clusters’ identification.

|  | Wheeze ever to age 5 yrs | Wheeze limiting speech | With exercise | Without exercise | With colds | Apart from colds | Shortness of breath | Chest tightness | Weather | Pollen | Flu | Dust | Pet | Fume | Emotions |
| --- | --- | --- | --- | --- | --- | --- | --- | --- | --- | --- | --- | --- | --- | --- | --- |
| NMI without | 0.75 | 1 | 0.97 | 0.96 | 0.94 | 0.97 | 0.90 | 0.88 | 0.97 | 0.98 | 0.94 | 1 | 1 | 0.98 | 1 |
| NMI individual | 0.62 | 0.114 | 0.27 | 0.396 | 0.51 | 0.276 | 0.37 | 0.42 | 0.31 | 0.23 | 0.50 | 0.17 | 0.12 | 0.11 | 0.13 |

References

1. Custovic A, Simpson BM, Murray CS, et al. The National Asthma Campaign Manchester Asthma and Allergy Study. *Pediatr Allergy Immunol* 2002; **13**(s15): 32-7.

2. Semic-Jusufagic A, Belgrave D, Pickles A, et al. Assessing the association of early life antibiotic prescription with asthma exacerbations, impaired antiviral immunity, and genetic variants in 17q21: a population-based birth cohort study. *Lancet Respir Med* 2014; **2**(8): 621-30.

3. Atkinson W, Harris J, Mills P, et al. Domestic aeroallergen exposures among infants in an English town. *Eur Respir J* 1999; **13**(3): 583-9.

4. Martindale S, McNeill G, Devereux G, Campbell D, Russell G, Seaton A. Antioxidant intake in pregnancy in relation to wheeze and eczema in the first two years of life. *American journal of respiratory and critical care medicine* 2005; **171**(2): 121-8.

5. Miller MR, Hankinson J, Brusasco V, et al. Standardisation of spirometry. *European respiratory journal* 2005; **26**(2): 319-38.

6. Beydon N, Davis SD, Lombardi E, et al. An official American Thoracic Society/European Respiratory Society statement: pulmonary function testing in preschool children. *Am J Respir Crit Care Med* 2007; **175**(12): 1304-45.

7. Reddel HK, Taylor DR, Bateman ED, et al. An official American Thoracic Society/European Respiratory Society statement: asthma control and exacerbations: standardizing endpoints for clinical asthma trials and clinical practice. *Am J Respir Crit Care Med* 2009; **180**(1): 59-99.

8. Deliu M, Fontanella S, Haider S, et al. Longitudinal trajectories of severe wheeze exacerbations from infancy to school age and their association with early‐life risk factors and late asthma outcomes. *Clinical & Experimental Allergy* 2020; **50**(3): 315-24.

9. McCarthy S, Das S, Kretzschmar W, et al. A reference panel of 64,976 haplotypes for genotype imputation. *Nat Genet* 2016; **48**(10): 1279-83.

10. MacQueen J. Some methods for classification and analysis of multivariate observations. Proceedings of the fifth Berkeley symposium on mathematical statistics and probability; 1967: Oakland, CA, USA; 1967. p. 281-97.

11. Forgey E. Cluster analysis of multivariate data: Efficiency vs. interpretability of classification. *Biometrics* 1965; **21**(3): 768-9.

12. Jancey R. Multidimensional group analysis. *Australian Journal of Botany* 1966; **14**(1): 127-30.

13. Lloyd S. Least squares quantization in PCM. *IEEE transactions on information theory* 1982; **28**(2): 129-37.

14. Rosenberg A, Hirschberg J. V-measure: A conditional entropy-based external cluster evaluation measure. Proceedings of the 2007 joint conference on empirical methods in natural language processing and computational natural language learning (EMNLP-CoNLL); 2007; 2007. p. 410-20.
